# Targeting EGR1-ATF3 signaling mitigates paravertebral muscle degeneration by regulating cell death and inflammaging

**DOI:** 10.1186/s40659-025-00634-1

**Published:** 2025-07-28

**Authors:** Xuke Wang, Qingfeng Wang, Zhe Wang, Yingjie Zhou, Xiaobing Jiang, Yongjin Li

**Affiliations:** 1 Department of Orthopaedics, The First Affiliated Hospital of Guangzhou University of Chinese Medicine, Guangzhou City, Guangdong Province 510405 China; 2 Department of Minimally Invasive Spine Surgery, Luoyang Orthopedic Hospital of Henan Province. Orthopedic Hospital of Henan Province, 82 Qiming South Road, Luoyang City, Henan Province 471000 China; 3 Spine Center, Department of Orthopaedics, The First Affiliated Hospital of USTC, Division of Life Sciences and Medicine, University of Science and Technology of China, No.17, Lujiang Road, Hefei City, Anhui Province 230001 China; 4 Department of Orthopedics, Xuanwu Hospital, Capital Medical University, No. 45 Changchun Street, Xicheng District, Beijing City, 230012 China

**Keywords:** Paravertebral muscle degeneration, EGR1-ATF3 signaling, Inflammaging, Cell death, RNA sequencing

## Abstract

The paravertebral muscles play a critical role in maintaining dynamic spinal stability and physiological function. With aging, these muscles undergo senescence and degeneration, contributing to spinal instability and the development of low back pain. Age-related cellular death further accelerates chronic, low-grade inflammation termed “inflammaging” and disrupts the extracellular matrix (ECM), representing a key pathogenic mechanism driving paravertebral muscle degeneration (PMD). However, the core regulatory genes orchestrating inflammaging in this context have yet to be fully elucidated. The paravertebral muscles play an important role in supporting dynamic stability and physiological function of the spine. This study identified 409 differentially expressed genes (DEGs) through RNA sequencing. Subsequent bioinformatics analysis revealed 81 functionally relevant DEGs, with several hub genes such as Activating Transcription Factor 3 (ATF3), Cyclin-Dependent Kinase Inhibitor 1 A (CDKN1A/p21), and Interleukin-6 (IL-6) being significantly upregulated. These genes are associated with cellular death, ECM metabolic dysregulation, and inflammaging. Functional experiments demonstrated that silencing ATF3 attenuated cellular death, reduced inflammatory signaling, and preserved ECM integrity by modulating downstream effectors including CDKN1A/p21, IL6, Gasdermin E (GSDME), and Glutathione Peroxidase 4 (GPX4). Further network analysis identified the Early Growth Response 1 (EGR1)–ATF3 signaling axis, with EGR1 knockdown protecting against PMD through downregulation of ATF3. These genes may also exhibit high specificity and sensitivity for distinguishing PMD, suggesting their potential utility as diagnostic biomarkers. Overall, this study provides new insights into the molecular mechanisms underlying PMD and offers promising targets for therapeutic intervention.

**Supplementary Information:**

The online version contains supplementary material available at 10.1186/s40659-025-00634-1.

## Introduction

Low back pain is a prevalent symptom of musculoskeletal disorders and represents a major contributor to the global diseases burden. It continues to pose significant challenges worldwide in terms of prevention, diagnosis, treatment, and rehabilitation [1–4]. According to the data from China’s Seventh National Population Census in 2020, the population aged 65 and above reached nearly 200 million, accounting for 13.5% of the country’s total population. This reflects a deepening trend of population aging, with a growing proportion of elderly individuals at risk. The paravertebral muscles, a collective term for the muscle groups surrounding the spine, play a critical role in maintaining movement and dynamic stability of the spine [1, 5]. As individuals age, these muscles progressively undergo cell senescence and structural degeneration. These two are interlinked in a self-perpetuating cycle: senescence increases susceptibility to degeneration, while degeneration further accelerates the aging of muscle tissue. This interplay leads to a progressive decline in muscle mass and function known as sarcopenia, thereby weakening spinal support, promoting instability, and ultimately contributing to the onset of low back pain [1, 5–7]. Takada et al. [6] showed that cell senescence promotes fatty infiltration and fibrosis in the rotator cuff muscles of mice, thereby accelerating muscle degeneration. Moreover, low back pain itself limits physical activity, which further intensifies muscle senescence and degeneration, creating a vicious cycle [1, 5]. Consequently, understanding the pathological mechanisms driving age-related paravertebral muscle degeneration (PMD) and interrupting this harmful feedback loop is essential for enhancing the quality of life in the aging population.

Chronic inflammation is a key driver and hallmark of aging and degeneration [1, 5, 8]. Aging is associated with a persistent, chronic low-grade inflammatory state—termed “inflammaging”, which plays a significant role in the development of numerous age-related degenerative conditions, including PMD [7–10]. The key pathological features of PMD include fatty infiltration, collagenous fibrosis, and loss of muscle mass [1, 5]. These degenerative changes are largely driven by elevated levels of pro-inflammatory cytokines, highlighting inflammation as a central mechanism in age-related muscle deterioration [7, 11, 12]. Inflammatory cell death (pyroptosis/ferroptosis) triggers reactive oxygen species (ROS) accumulation and Interleukin-6/ Interleukin-1 beta (IL-6/IL-1β) production, promoting inflammaging, extracellular matrix (ECM) breakdown, contributing to PMD [1, 9, 13–15]. We propose that pro-inflammatory factors initiate inflammatory cell death, leading to a sustained release of cytokines and chronic inflammation. This process impairs ECM synthesis, accelerates fat accumulation, muscle atrophy, and fibrosis, ultimately initiating or exacerbating PMD. Therefore, there is an urgent need to identify the key cellular and molecular drivers of PMD to develop targeted interventions.

.Muscle stem cells (MuSCs) possess the ability to both self-renew and differentiate into myofibers, playing a vital role in muscle regeneration and repair [16]. Building on our previous single-cell genomic profiling efforts, we have identified a distinct resident population of MuSCs within the paravertebral muscles [17]. Targeted gene therapy aimed at modulating endogenous MuSCs holds promise for improving the local microenvironment, halting degenerative pathways, expanding the functional MuSC pool, and ultimately enhancing muscle regeneration [18]. However, the key gene regulatory networks governing MuSC behavior in this context have yet to be comprehensively defined.

RNA sequencing is a widely used technique for identifying differentially expressed genes (DEGs) in tissues and uncovering their biological and underlying mechanisms [15, 19, 20]. To investigate the transcriptional landscape of PMD, human paravertebral muscle samples were analyzed using RNA sequencing. This study aims to identify key DEGs and pathways involved in PMD, with a particular emphasis on their roles in regulating cell death and inflammaging. The findings are intended to lay the groundwork for biomarker discovery and the development of targeted, precision therapies for PMD.

## Materials and methods

### Patient recruitment

This study was conducted with ethical approval from the Luoyang Orthopedic Hospital of Henan Province and the Capital Medical University Xuanwu Hospital Ethics Committee. For RNA sequencing, paravertebral muscle tissues were collected from three individuals with relatively normal muscle condition and three with severe degeneration. Additionally, 10 patients diagnosed with PMD and 10 individuals without PMD were recruited for qRT-PCR validation. All participants provided written informed consent prior to participation. The cross-sectional area of muscle is a key parameter for assessing paravertebral muscle health, with a larger area indicating better muscle quality. We selected the L4/L5 segment of the lumbar spine for sample collection due to its relatively large average muscle cross-sectional area, ease of measurement, and its frequent use in paravertebral muscle research [15, 17, 21]. The severity of PMD was evaluated using the Goutallier grading system, which provides a visual, semi-quantitative assessment of fat infiltration [17, 21, 22]. Individuals with Goutallier grades 0–1 were classified as the normal group, while those with grade 4 were assigned to the severe PMD (SPMD) group.

### Muscle digestion

The paravertebral muscle tissues were placed in sterile phosphate buffer saline supplemented with 4% streptomycin (Gibco Thermofish). The tissues were washed twice with the same solution to ensure cleanliness. The visible connective tissue, blood vessels, and adipose tissue were then carefully removed. The cleaned muscle samples were then enzymatically digested using a mixture of Collagenase II, Dispase II, and a red blood cell lysis reagent [15, 17].

### RNA sequencing and data analysis

The total RNA was extracted from adult paravertebral muscle tissues using the TRIzol reagent (Life Technologies, USA). The purity and integrity of RNA were assessed using a NanoPhotometer spectrophotometer and an Agilent 2100 bioanalyzer. RNA library preparation was carried out following the manufacturer’s instructionsusing the NEBNext^®^ Ultra™ RNA Library Prep Kit. RNA sequencing was performed by NovelBio Biopharmaceutical Technology Company. After data quality control, the gene expression was quantified using FPKM values with a detection threshold of FPKM ≥ 0.5 in ≥ 70% of samples per group; miRNA expression required normalized counts ≥ 5 in ≥ 50% of samples. The differentially expressed genes (DEGs) and differentially expressed miRNAs (DEMs) were analyzed using R software EBSeq package with statistical significance defined as|log_2_ fold-change (FC)| >1 and false discovery rate (FDR) < 0.05. Data visualization, including cluster heatmaps, volcano plots, and Sankey diagrams were output using R software pheatmap package, enhancedVolcano package, and networkD3 package, respectively [23].

### Identification of the functional DEGs in PMD

Gene sets related to the extracellular matrix, oxidative stress, and ferroptosis were obtained from studies by Li et al. [19, 20], while genes associated with. autophagy, cell senescence, inflammasomes, pyroptosis, and apoptosis were sourced from Qiu et al. [24]. These genes were listed in Supplementary Table S1. To identify the functional DEGs associated with PMD, Venn plots were used to merge these genes with DEGs obtained from RNA-seq to screen for overlapping genes.

### Protein-protein interaction (PPI) analysis

Search tool for recurring instances of neighbouring genes (STRING) database (https://string-db.org/) was used to build a PPI network for functional differentially expressed genes [25]. First, the DEGs were entered into the STRING database to obtain the interaction network and associated data files. These data were then imported into the Cytoscape software for visualization and further analysis. Key hub genes were subsequently identified using five algorithms (maximum clique centrality (MCC), Degree, Betweenness, EPC, and Closeness) available through the CytoHubba in Cytoscape [26]. Genes consistently ranked in the top 10 by all methods were defined as high-confidence hubs.

### Gene ontology (GO) and Kyoto encyclopedia of genes and genomes (KEGG) pathway enrichment analysis

GO resource (http://geneontology.org) is an internationally standardized gene function classification database, encompassing three major categories: biological processes (BP), cell components (CC), and molecular functions (MF). It provides structured, computable information about gene and gene product functions [27]. KEGG (https://www.genome.jp/kegg/) pathway plays an crucial role in the gene annotation process, helping to identify the signaling pathways in which genes are involved [28]. The cutoff was defined as p-value < 0.05. BP and KEGG can directly reflect the potential function of genes. We used the R software clusterProfiler package (v4.0) for GO and KEGG enrichment analysis.

### Construction of the DEMs-hub DEGs signaling network in PMD

For the key functional hub DEGs identified above, the upstream miRNAs of them were predicted using mirDIP 5.2 version (https://ophid.utoronto.ca/mirDIP/index.jsp) database [29]. PMD-related DEMs were then determined by intersecting the DEMs obtained from RNA sequencing with the predicted upstream miRNAs of each hub DEG from mirDIP. Based on these results, a comprehensive DEM–hub DEG regulatory network was constructed using Cytoscape software [26].

### Transcription factor (TF)-gene regulatory network construction

NetworkAnalyst 3.0 database (https://www.networkanalyst.ca/) is a visual analytics tool for comprehensive gene expression profiling [30]. TF–target gene interaction data were sourced from the JASPAR database (http://jaspar.genereg.net/), which is integrated into the NetworkAnalyst 3.0 platform [31]. The TF-gene co-regulatory network was constructed in NetworkAnalyst 3.0 by uploading the top 10 hub DEGs (|log2FC| >1, *p* < 0.05) into the gene list input field with ‘Homo sapiens’ selected as the species. JASPAR database (TF-motif predictions, p < 1 × 10^-^⁵) was used under ‘TF-gene interactions’, and nodes were filtered by Degree > 1 and Betweeness > 0 to prioritize biologically relevant connections. The resulting network was then visualized using Cytoscape software [26].

### Predicting the prognostic value of the key hub DEGs and DEMs

Receiver Operating Characteristic (ROC) curve and Precision Recall (PR) curve are commonly used to predict accuracy. In this study, these analyses were performed using the pROC package and ggplot2 packages in R software. The diagnostic potential of key DEGs was evaluated based on the area under the curve (AUC), with values closer to 1, indicating stronger predictive performance.

### Extraction and cultivation of primary muscle stem cells (MuSCs)

The MuSCs were isolated enzymatically based on the instructions described [32]. Briefly, human paravertebral muscle were obtained from lumbar disc herniation patients who undergo spinal surgery. The muscle was then mechanically disrupted into pieces using sterile scissors, and followed by digesting with 0.03 g of 0.2% collagenase II (Worthington, LS004176) supplemented with 4% penicillin‒streptomycin and 15 ml DMEM medium. This was followed by a second digestion step using an additional 15 mL of DMEM (Gibco, Cat# 11965092), supplemented with 0.04 g of 0.2% collagenase II, 2 ml of trypsin, 2 ml of DNase (1 mg/ml), and 1% penicillin-streptomycin were added to the digestion mixture for further digestion. After enzymatic digestion, the cell suspension was filtered through a 300-mesh nylon filter, and centrifuged at 350 × g for 5 min. The isolated MuSCs were then cultured in Ham’s F10 medium (CellWorld, C1662-875) supplemented with 20% fetal bovine serum (FBS), 1% penicillin‒streptomycin (Sigma, P4333), and 5 ng/ml basic fibroblast growth factor (PeproTech #100-18B) [33]. The culture dishes were coated with Matrigel (Corning, 354230). MuSCs cultures were placed in 37 °C incubators with 5% CO_2_.

### RNA extraction and quantitative real-time RT-PCR

We used the TRlzol Reagent (Life Technologies, USA) to extract total RNAs from paravertebral muscle tissues. RNA purity and concentration were assessed using a microspectrophotometer (Allsheng, China), while RNA integrity was confirmed via gel electrophoresis. Complementary DNA (cDNA) was synthesized from the extracted RNA using the one Step PrimeScript™RT-PCR Kit (Takara, Japan). qRT-PCR was then carried out using the Thermal Cycler Dice™ Real-Time System III (Takara, Japan). After the qRT-PCR reaction, both melting curves and amplification curve were examined to confirm the specificity and efficiency of the amplification. The forward primers and reverse primers of each gene used in this study are listed in Supplementary Table S2. Their relative expression levels were measured using the 2^−△△^Ct method, as described previously [14, 19].

### Western blotting

Total protein was extracted from normal and degenerative paravertebral muscle tissues using radioimmunoprecipitation assay (RIPA) lysis buffer along with a total protein extraction kit (Bestbio, Shanghai, China; Catalog #BB-3101) following the manufacturer’s protocol. The protein concentration was measured using the Micro Bicinchoninic Acid Protein (BCA) Assay Kit (Beyotime, China), which allows for accurate quantification of total protein content. Proteins were separated by Sodium Dodecyl Sulfate–Polyacrylamide Gel Electrophoresis (SDS-PAGE) and subsequently transferred onto a Polyvinylidene Difluoride (PVDF) membrane (Merck, Germany). These membranes were incubated overnight at 4 °C with primary antibodies anti-ATF3 (ab207434, Abcam), anti-Aggrecan (ab3778, Abcam), anti-CDKN1A/P21 (ab102013, Abcam), anti-GPX4 (ab125066, Abcam), anti-Caspase3 (ab32351, Abcam), anti-GSDME (ab215191, Abcam), and β-actin (Proteintech, 66009-1-1 g) in 5% non-fat dry milk in PBS containing 0.1% Tween-20. The PVDF membranes were washed and incubated with the appropriate secondary antibody for 1 h at room temperature. Protein bands were detected using enhanced chemiluminescence (ECL) reagents (Beyotime, Shanghai, China) and visualized with the ChemiDoc™Touch Imaging System (Bio-Rad, Berkeley, CA, USA).

### Detection of cell death

Cell death rates was measured by conducting flow cytometry analysis of cells stained with Annexin V and propidium iodide (PI). The VF647A-Annexin V/PI Apoptosis Detection Kit (HY-K1093-50T, MedChemExpress) was used to for this purpose. The treated cells were collected, washed with PBS, and stained with Annexin V-FITC and PI in the dark at room temperature for 15 min. The stained samples were then analyzed using a flow cytometer (BD FACSAriaTM II). Subsequently, FlowJo VX10 software was used to analyze the data. In flow cytometry apoptosis assays, Q2 typically indicates late apoptosis (Annexin V^+^/PI^+^), while Q3 indicates early apoptosis (Annexin V^+^/PI^-^). The sum of Q2 and Q3 is commonly used to quantify overall apoptosis.

### Determination of glutathione (GSH), ROS, and fe^2+^ expression level

Based on the manufacturer’s protocols, GSH assay kit (CS0260, Sigma) and iron assay kit (Abcam, Cambridge, UK) were used to assess the relative GSH and iron concentrations in cell lysates. Intracellular ROS levels were detected using the oxidation-sensitive fluorescent probe DCFH-DA, following a previously described method. The analysis was performed with a ROS Assay Kit (Beyotime, China) and quantified via flow cytometry using the FACSCalibur system (BD Biosciences).

### Enzyme-linked immunosorbent assay (ELISA)

Cell lysis were harvested following 24 h after transfection. According to the manufacturer’s protocols, human IL-1β and IL-6 ELISA kit (Elabscience, China) were used to measure the intracell concentrations of IL-1β and IL-6 under various treatment conditions. Briefly, 96-well plates were coated with 100 µL/well of capture antibody and incubated overnight at 4 °C. After washing (3× with wash buffer), plates were blocked with 300 µL/well of reagent diluent for 1 h at room temperature. IL-1β and IL-6 standards and normalized samples (50 µg total protein/well) were added in duplicate (100 µL/well) and incubated for 2 h at room temperature. Plates were incubated with detection antibody 1 h, and followed by streptavidin-HRP 30 min at room temperature. Absorbance was measured at 450 nm (correction at 570 nm) using a microplate reader (BioTek Synergy H1). IL-1β and IL-6 concentrations were calculated from standard curves using 4-parameter logistic regression.

### Dual-Luciferase reporter assay

The binding site between EGR1 and ATF3 promoter was predicted using JASPAR database (https://jaspar.elixir.no/) [31]. pGL4.74[hRluc/TK] (Promega, backbone: Renilla luciferase) was used for normalization. The ATF3 promoter region (wild-type and mutant) was cloned into the pGL4.10[luc2] vector (Promega, backbone: Firefly luciferase). The ATF3 wild-type and mutant promoter were amplified by PCR from genomic DNA, the purified PCR products were ligated into the pGL4.10[luc2] vector and T4 DNA ligase. All constructs were verified by sanger sequencing. Full-length human EGR1 cDNA was subcloned into the pcDNA3.1(+) vector (Thermo Fisher Scientific) to overexpress EGR1. EGR1 siRNA was used to knockdown EGR1. The luciferase reporter vectors and EGR1 overexpression vector/siRNA were co-transfected to human embryonic kidney (HEK) 293T cells using Lipofectamine 8000 (Beyotime, China). Luciferase activity was measured using the Luciferase Assay Kit and GloMax^®^ 20/20 Luminometer (Promega, Madison, WI, USA). Reporter gene activation was assessed by normalizing Firefly luciferase relative light units (RLU) to to Firefly luciferase RLU in each sample.

### Statistical analysis

Statistical analysis of the quantitative data was performed using GraphPad Prism version 8. For data following a normal distribution, group comparisons were conducted using an unpaired Student’s *t-*test. If data violated normality assumptions, Mann-Whitney U test (unpaired) or Wilcoxon signed-rank test (paired) were used to analyze data. Results are expressed as mean ± standard deviation (SD). The *P*-value less than 0.05 represented statistical significance.* represents *p* < 0.05; ** represents *p* < 0.01 and *** represents *p* < 0.001.

## Results

### Identification and analysis of the gene and miRNA expression profiles in PMD using RNA sequencing

**Fig. 1:**
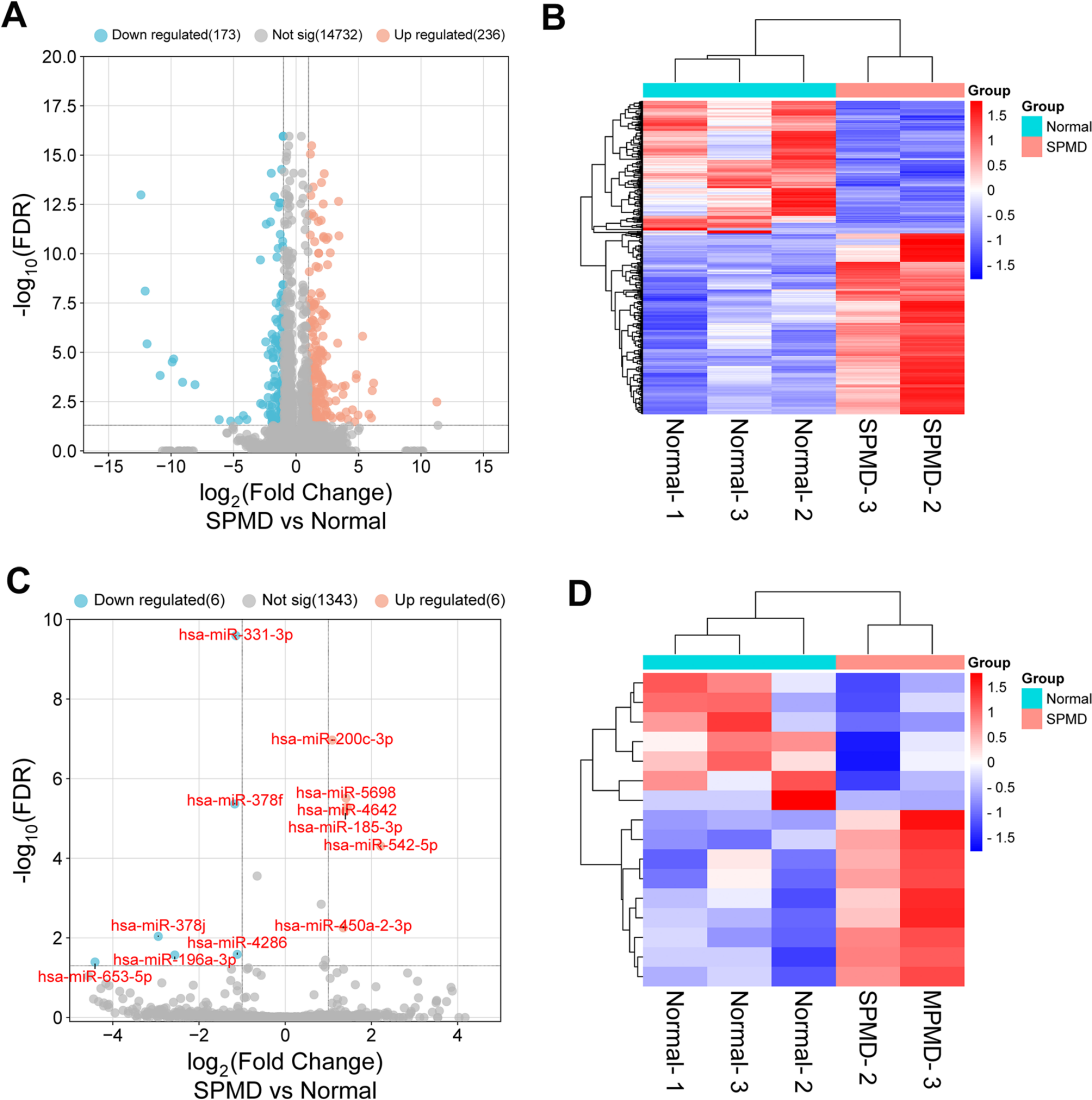
Identification of the mRNA and miRNA expression profile in PMD by RNA-seq. **A**: Volcano Plot displaying the mRNA expression profile in PMD. The vertical axis represents the FDR, and the horizontal axis represents the fold change. Red dots represents upregulated genes, blue dots represents downregulated genes, and gray dots represent genes that were not differentially expressed. **B**: Cluster heatmap displaying the differentially expressed mRNAs in PMD. The vertical axis represents the differentially expressed mRNAs, and the horizontal axis represents samples. **C**: Volcano Plot displaying the miRNA expression profile in PMD, where the differentially expressed miRNAs are labeled. **D**: Cluster heatmap displaying the differentially expressed miRNAs in PMD

In our previous study, we validated the quality of paravertebral muscle tissue samples using hematoxylin-eosin and Masson’s trichrome staining [15, 17]. For RNA sequencing, we initially selected three relatively normal and three severely degenerated paravertebral muscle samples. However, one of the degenerated muscle samples exhibited abnormalities and significant transcriptomic divergence from the others, suggesting widespread cell death within that tissue [15, 17]. In contrast, the remaining samples demonstrated compositional consistency [15, 17]. Therefore, we proceeded with the analysis using three normal and two severely degenerated muscle samples. RNA sequencing data was processed using the EBSeq package in R. Using these stringent thresholds, we identified 15,141 expressed genes (FPKM ≥ 0.5) and 1,355 detectable miRNAs (normalized counts ≥ 5) in PMD samples. Of these, 409 DEGs and 12 DEMs showed significant differential expression (| log_2_ (FC)|>1 and FDR < 0.05) compared to controls (Fig. 1A and C). Among these, 137 DEGs were significantly downregulated and 236 DEGs were significantly upregulated (Fig. 1A-D). Based on these data, we observed miR-653-5p and miR-542-5p were the most significantly downregulated and upregulated DEMs in PMD (Supplementary Table S3).

### Identification of the key functional hub DEGs in PMD

**Fig. 2:**
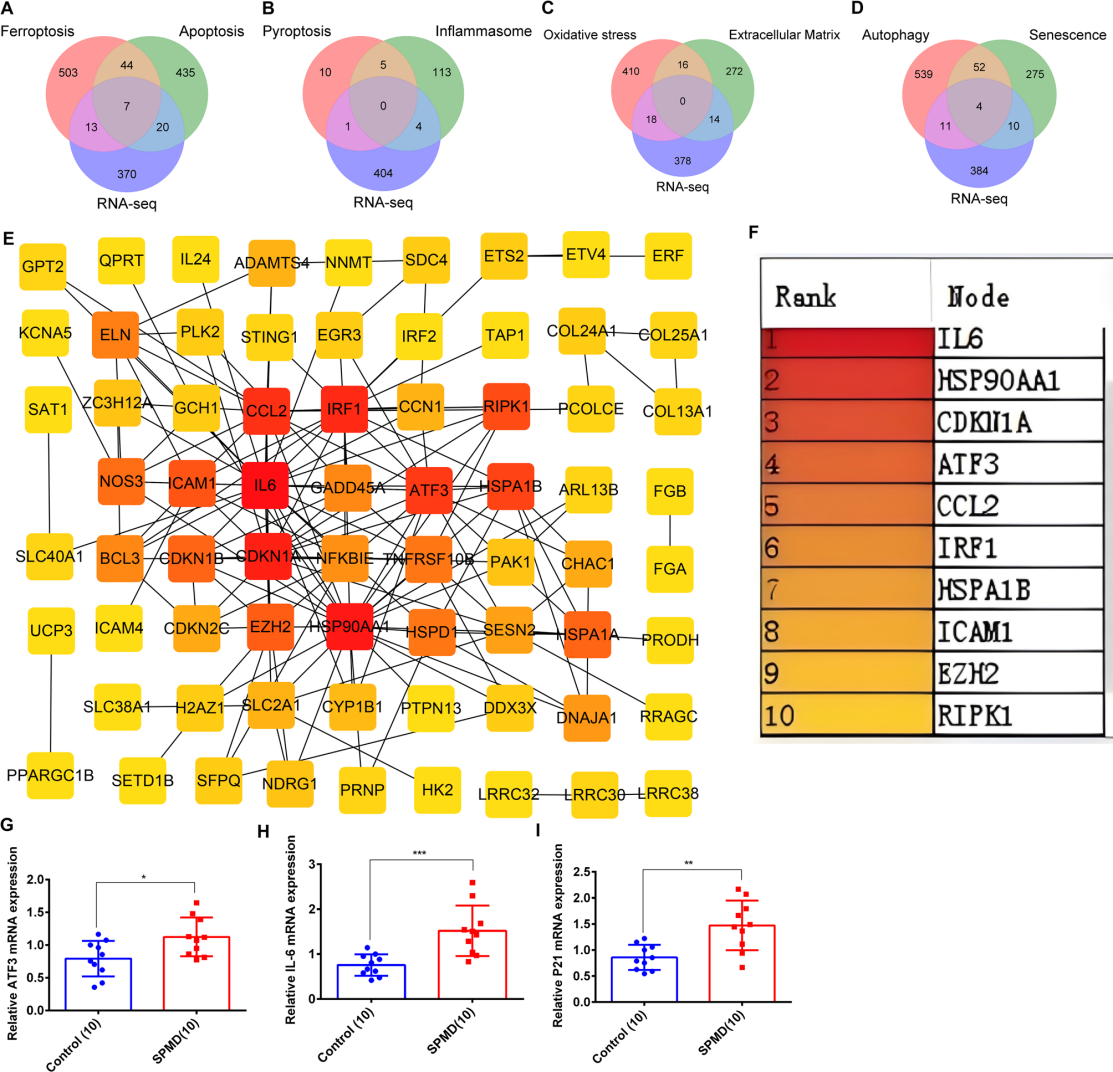
Screening the functional differentially expressed hub genes in PMD. **A**: Screening the differentially expressed PMD-related ferroptosis and apoptosis genes by the intersection of ferroptosis, apoptosis, and RNA-seq using Venn diagram analysis. **B**: Screening the differentially expressed PMD-related pyroptosis and inflammasome genes by the intersection of pyroptosis, inflammasome, and RNA-seq. **C**: Screening the differentially expressed PMD-related oxidative stress and extracellular matrix genes by the intersection of oxidative stress, extracellular matrix, and RNA-seq. **D**: Screening the differentially expressed PMD-related autophagy and senescence genes by the intersection of autophagy, senescence, and RNA-seq. **E**: A total of 81functional differentially expressed genes were imputed into the STRING database to produce a string interaction file and then was imputed into the Cytoscape software to construct a PPI regulatory network. **F**: CytoHubba plugin screened the top 10 hub genes using MCC algorithm. **G-I**: qRTPCR experiments were performed to detect the mRNA expression of IL-6, CDKN1A/P21, and ATF3 in normal and severe muscle samples. * represents *p* < 0.05; ** represents *p* < 0.01 and *** represents *p* < 0.001

Ferroptosis, pyroptosis, apoptosis, cell senescence, autophagy, oxidative stress, ECM imbalance, and inflammation are hallmark pathological features of PMD [15, 34]. The genes involved in regulating these processes are collectively referred to as functional genes. We merged these functional genes and DEGs obtained from RNA-seq, and identified 81 PMD-related functional DEGs (Fig. 2A-D and Supplementary Table S4).

To identify the key hub genes involved in PMD, a total of 81 functional DEGs were submitted to the STRING database to construct a PPI regulatory network (Fig. 2E). In Cytoscape using the CytoHubba plugin, the top 10 key hub genes were screened through five algorithms (MCC, Degree, Betweenness, EPC, and Closeness), namely (ATF3, CDKN1A, IL6, IRF1, HSPA1B, CCL2, ICAM1, HSP90AA1, RIPK1, and EZH2) (Fig. 2F and Supplementary Figure S1). Among these, four major hub genes were identified: Activating Transcription Factor 3 (ATF3), Cyclin-Dependent Kinase Inhibitor 1 A (CDKN1A/p21), IL-6, and Heat Shock Protein 90 Alpha Family Class A Member 1 (HSP90AA1). (Fig. 2F). Moreover, validation through quantitative real-time polymerase chain reaction (qRT-PCR) confirmed that the mRNA expression levels of IL-6, ATF3, and CDKN1A/P21 were significantly elevated in PMD samples, consistent with the RNA sequencing results. (Fig. 2G-I). Taken together, these findings suggest that ATF3, CDKN1A/P21, IL6 are key regulatory hub genes that modulate multiple pathological mechanisms in PMD, including ferroptosis, apoptosis, pyroptosis, cell senescence, ECM degradation, and inflammation.

### Exploration of the biological characteristics and functions of the key functional DEGs

**Fig. 3:**
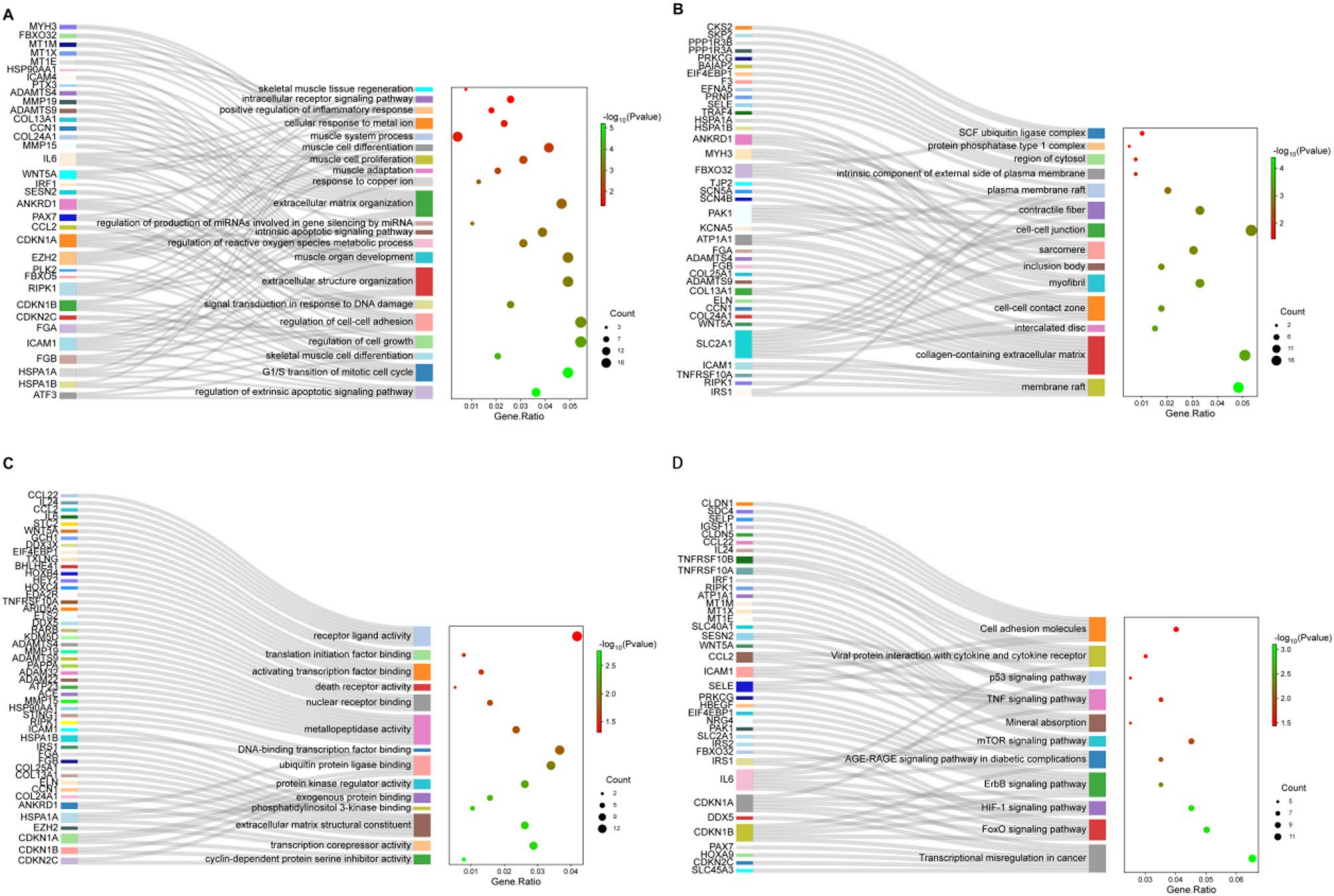
Go functional annotation and KEGG pathway enrichment analysis of the functional DEGs. Sankey and bubble diagram. The left is a sankey diagram, indicating the DEGs linked to each pathway; the right is a bubble diagram, the bubble size indicates the number of DEGs corresponding to each pathway, and the bubble color indicates the p value. **A**: Enrichment results of biological processes in GO analysis. **B**: Enrichment results of cell components in GO analysis. **C**: Enrichment results of molecular functions in GO analysis. **D**: Enrichment results of KEGG pathway

To determine the potential biological functions of 81 DEGs, functional enrichment analysis was performed. This analysis revealed significant enrichment in 782 biological processes (BP), 47 cellular components (CC), 64 molecular functions (MF), and 28 Kyoto Encyclopedia of Genes and Genomes (KEGG) pathways (*P* < 0.05). The key functional terms associated with ATF3 included skeletal muscle cell differentiation and muscle organ development. IL-6 was involved in mediating the inflammatory response, oxidative stress, and reactive oxygen species (ROS) production, while cell growth, cell cycle, ROS metabolic process were linked to CDKN1A. Both IL-6 and CDKN1A participate in multiple signaling pathways, including FoxO, hypoxia-inducible factor-1 (HIF-1), ErbB, mechanistic target of rapamycin (mTOR), tumor necrosis factor (TNF), and p53 (Fig. 3). Notably, ATF3, IL-6, and CDKN1A all regulate apoptosis and ferroptosis signaling pathways. Other genes are associated with extracellular matrix organization and cellular response to metal ions(Fig. 3). Importantly, an unbiased GO overrepresentation analysis was conducted on all 409 DEGs in the relevant comparison, with results concordant with the findings presented above (Supplementary Figure S2). Collectively, these findings suggest that ATF3, CDKN1A/p21, and IL-6 play critical roles in the pathogenesis of PMD by orchestrating multiple pathological processes.

### Construction of transcription factor (TF)-DEGs regulatory network in PMD

**Fig. 4:**
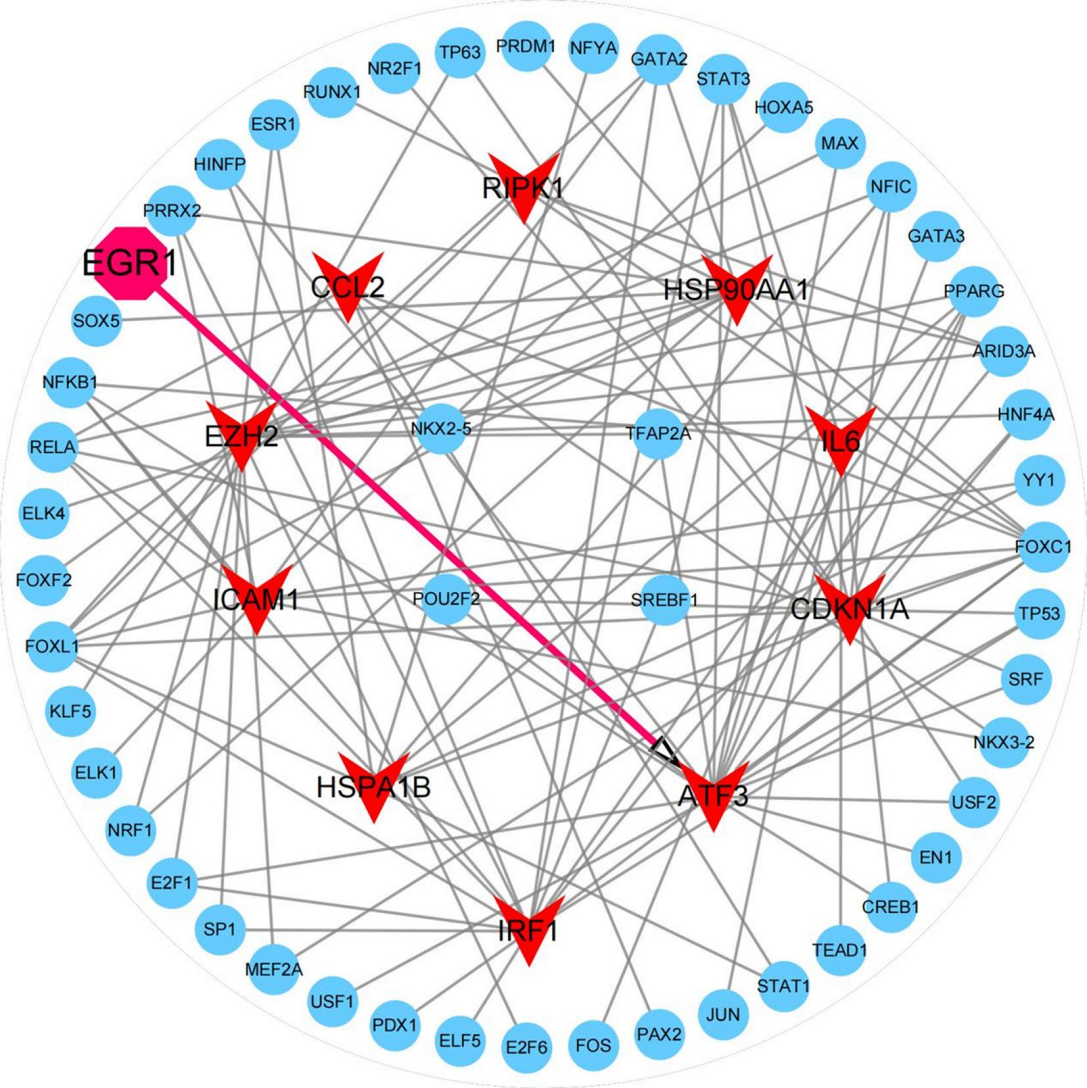
The regulatory network of transcription factors interacting with 10 key hub genes. The highlighted red nodes represented the 10 key hub genes and the other blue nodes represent transcription factors. The network consisted of 10 core genes, 61 nodes and 112 edges

According to the 10 key hub genes (ATF3, CDKN1A, IL6, IRF1, HSPA1B, CCL2, ICAM1, HSP90AA1, RIPK1, EZH2) (Fig. 2F), TFs regulating their expression were identified using the JASPAR TF database [31]. A TF-gene co-regulatory network was then constructed using the NetworkAnalyst 3.0 platform [30]. As illustrated in Fig. 4, resulting network comprised 10 seed genes, 61 nodes, 112 edges, and 51 transcription factors. Among these, ATF3 exhibited the highest centrality, with a Degree value of 19 and a Betweenness value of 462.3318, indicating regulation by 19 transcription factors, including Early Growth Response 1 (EGR1). Our single-cell transcriptomic analysis further revealed a notable co-upregulation of EGR1 and ATF3 in both PMD specimens and resident MuSCs, suggesting that the EGR1-ATF3 signaling is activated during PMD progression [17]. However, the specific functional roles and regulatory mechanisms of this signaling axis in PMD remain to be fully elucidated.

### ATF3 was identified as a target gene of EGR1

**Fig. 5:**
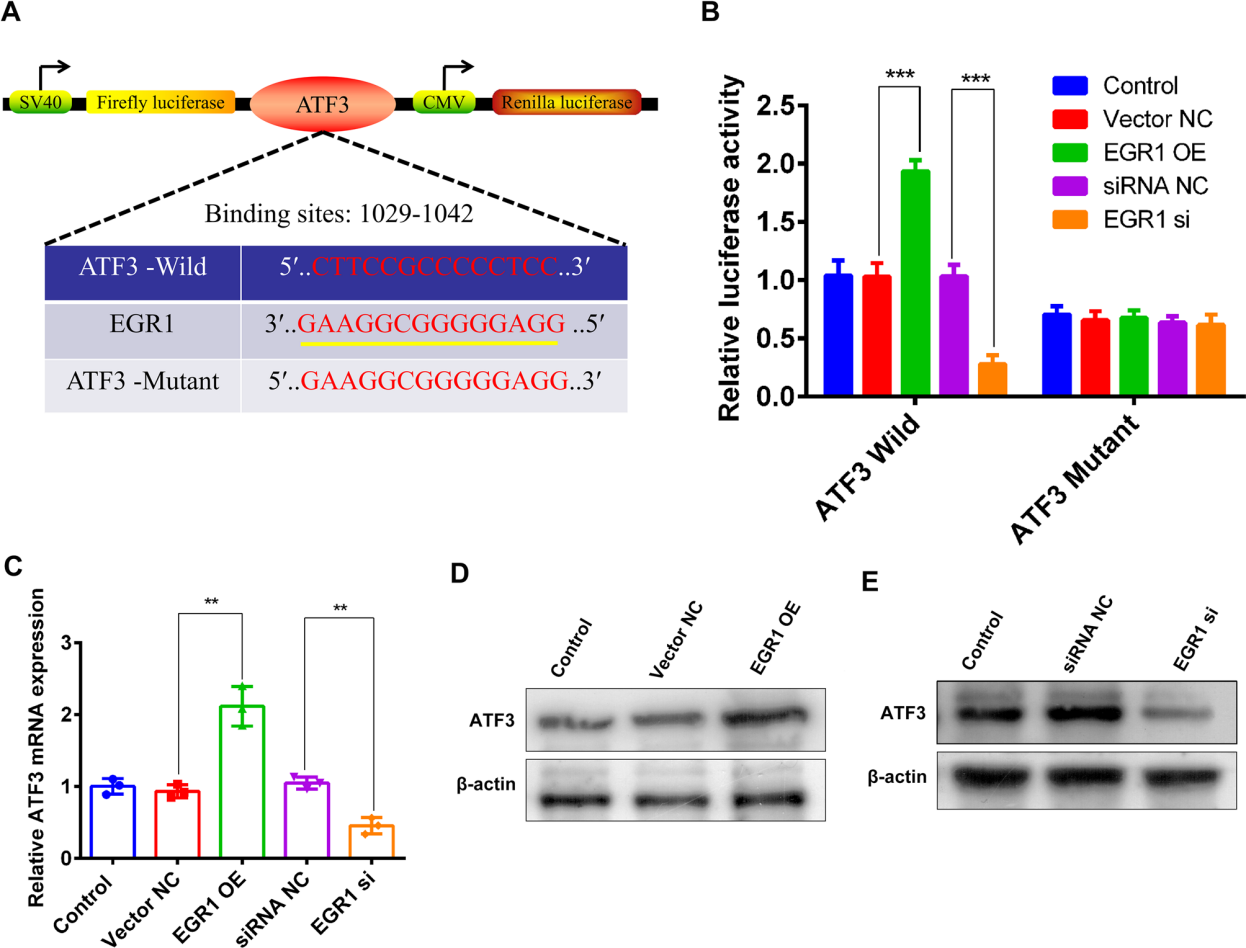
Validation of the interaction between ATF3 and EGR1. **A**: putative binding sites between EGR1 and ATF3 promoter were predicted using the JASPAR database. **B**: luciferase reporter assays demonstrated that EGR1 overexpression/knockdown significantly altered wild-type (WT) ATF3 luciferase activity, but had no effect on mutant-type (Mut) ATF3 activity. **C**: the mRNA expression of ATF3 cells was detected by qRT-PCR following EGR1 overexpression/knockdown. **D-E**: the protein expression of ATF3 in cells was examined by Western blotting after EGR1 overexpression/knockdown. ^∗∗^*P* < 0.01

To validate whether EGR1 directly binds to the ATF3 promoter, we performed luciferase reporter assays. Using JASPAR-predicted EGR1 binding sites (Fig. 5A), we cloned the wild-type (WT) ATF3 promoter and a mutant (Mut) version lacking the EGR1-binding motif. Luciferase reporter assays demonstrated that overexpression or knockdown of EGR1 significantly altered the luciferase activity of wild-type (WT) ATF3 promoter constructs. In contrast, no significant effect was observed in mutant-type (Mut) constructs, indicating that EGR1 directly bound to and regulated the ATF3 promoter. (Fig. 5B), suggesting that EGR1’s direct binding to the ATF3 promoter. Furthermore, in MuSCs, ectopic EGR1 expression of EGR1 markedly increased both ATF3 mRNA and protein levels, while EGR1 silencing led to a significant reduction in ATF3 expression (Fig. 5C and D). Collectively, these findings provide compelling evidence that EGR1 directly regulates ATF3, uncovering a previously unrecognized EGR1–ATF3 signaling axis involved in the pathogenesis of PMD.

### Silencing of ATF3 prevented PMD by regulating muscs death and inflammaging

**Fig. 6:**
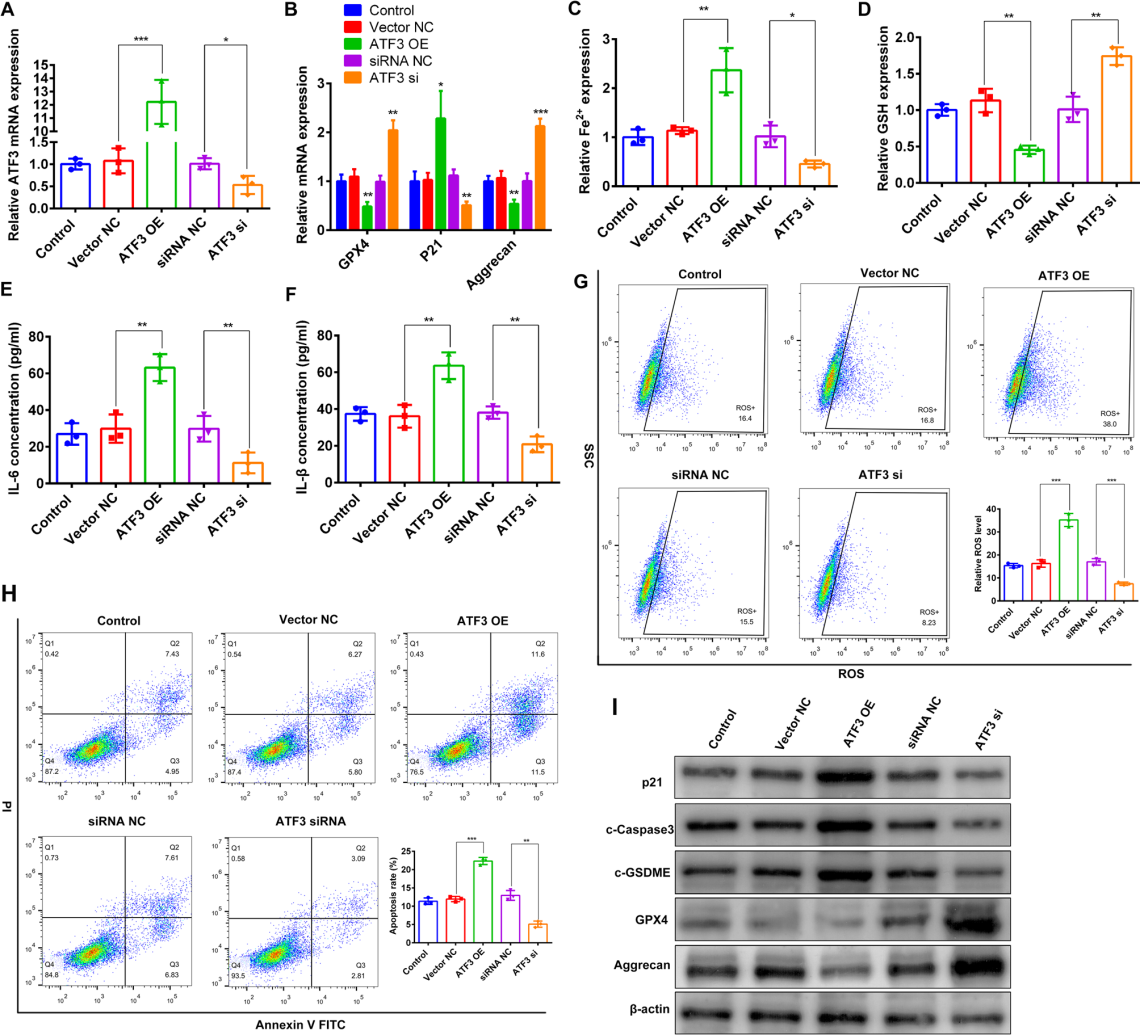
Validation of ATF3’s role in PMD. **A**: qRT-PCR analysis of the effects of ATF3 overexpression/knockdown. **B**: mRNA levels of the ferroptosis-related gene GPX4, senescence marker p21, and extracellular matrix component Aggrecan were measured by qRT-PCR in MuSCs following ATF3 overexpression/knockdown. **C-D**: Intracell Fe^2+^ and glutathione (GSH) levels in MuSCs after ATF3 overexpression/knockdown. **E-F**: IL-6 and TNF-α concentrations in MuSCs were quantified by ELISA upon ATF3 overexpression/knockdown. **G-H**: Flow cytometry analysis of ROS levels and apoptosis rates in MuSCs after ATF3 overexpression/knockdown. **I**: Western blot detection of multiple protein markers following ATF3 overexpression/knockdown. * represents *p* < 0.05; ** represents *p* < 0.01 and *** represents *p* < 0.001

To determine the biological significance of the EGR1-ATF3 signaling axis in the pathogenesis of PMD, we performed both gain- and loss-of-function experiments in MuSCs. Overexpression of ATF3 in MuSCs led to a significant reduction in both mRNA and protein levels of Glutathione Peroxidase 4 (GPX4), a key marker of ferroptosis and Aggrecan, a major component of the extracellular matrix. Conversely, there was a marked upregulation of pro-inflammatory cytokines IL-6 and IL-1β, CDKN1A/p21, pyroptosis-associated protein Gasdermin E (GSDME), and the apoptosis marker Caspase-3.(Fig. 6A, B,E, F,I). ATF3 overexpression exacerbated iron overload, enhanced ROS production, and increased cell mortality, accompanied by depleted intracellular glutathione GSH content. All these pathological changes were notably mitigated when ATF3 expression was knocked down (Fig. 6C, D,G, H). It remains a compelling topic for future research whether ATF3 directly binds to the promoters of GPX4 and GSDME or indirectly regulates them via intermediate factors. Taken together, these findings demonstrate that ATF3 plays a central role in promoting inflammatory cell death and inflammaging, thereby contributing to the progression of PMD..

### Blocking EGR1-ATF3 signaling mitigated PMD progression

**Fig. 7:**
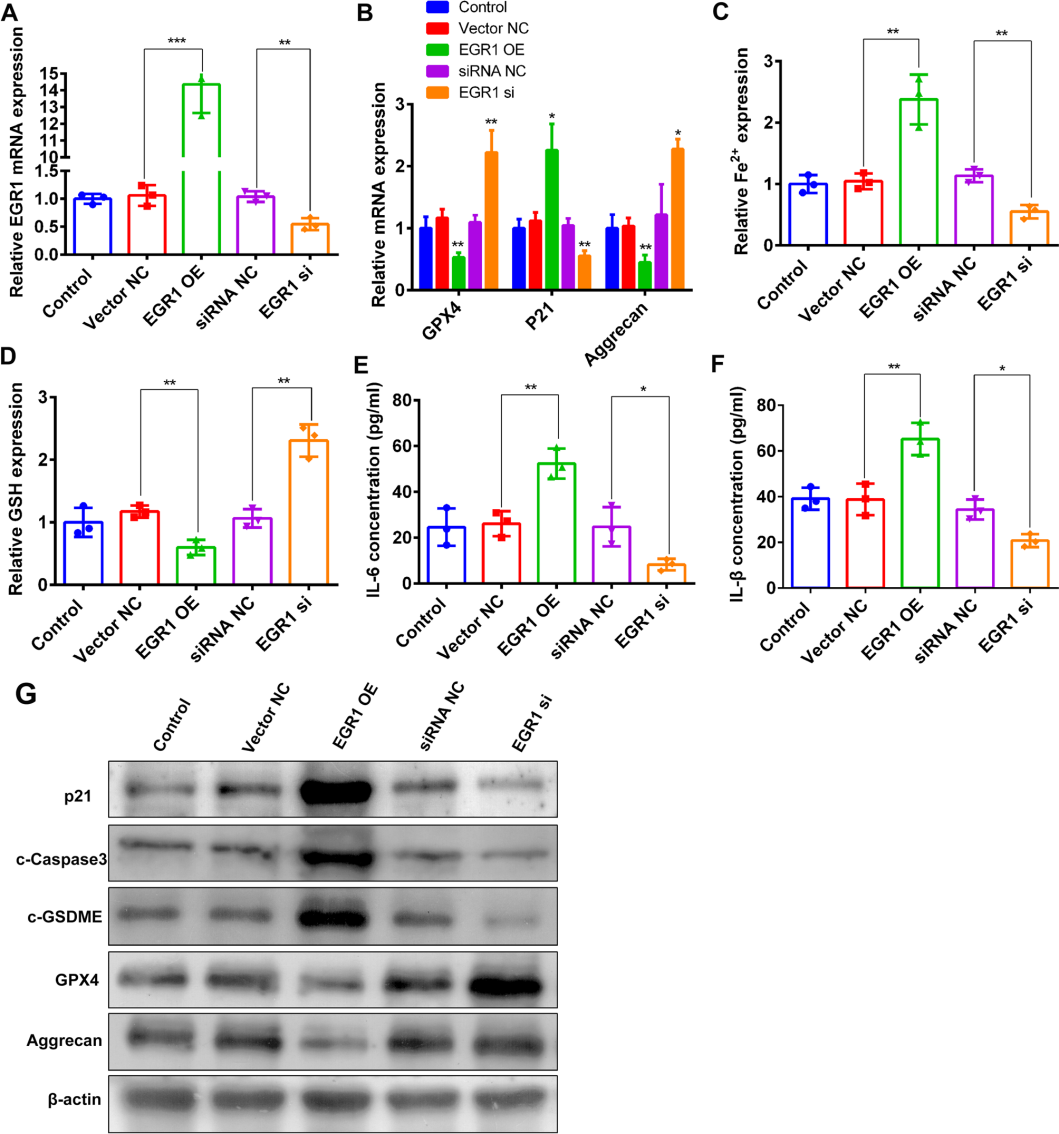
Functional validation of EGR1 in PMD. **A**: qRT-PCR analysis of EGR1 overexpression/knockdown efficiency. **B**: the mRNA expression levels of ferroptosis-related gene GPX4, senescence marker p21, and extracellular matrix component Aggrecan were examined by qRT-PCR in MuSCs following EGR1 modulation. **C-D**: Intracell Fe^2+^ and glutathione (GSH) levels were measured after EGR1 overexpression/knockdown. **E-F**: IL-6 and TNF-α secretion levels were quantified by ELISA upon EGR1 manipulation. **G**: Protein expression profiles of key markers were analyzed by Western blot after EGR1 overexpression/knockdown. * represents *p* < 0.05; ** represents *p* < 0.01 and *** represents *p* < 0.001

Transfection with EGR1-expressing plasmids robustly elevated EGR1 levels in MuSCs, whereas EGR1-targeting siRNA achieved efficient gene silencing (Fig. 7A). In line with the downstream regulatory role of ATF3, EGR1 overexpression led to a coordinated activation of multiple pathological processes: (i) promotion of ferroptosis, evidenced by iron overload and decreased levels of GPX4 and GSH; (ii) activation of pyroptosis and apoptosis, indicated by upregulated expression of GSDME and Caspase3; (iii) enhancement of inflammaging phenotypes with elevated levels of IL-6, IL-1β, and CDKN1A/p21; and (iv) disruption of ECM homeostasis, marked by reduced Aggrecan expression (Fig. 7B-G). Notably, silencing of EGR1 fully reversed these pathological changes, identifying EGR1 as a key upstream regulator of this degenerative network (Fig. 7B-G). These findings highlight the EGR1–ATF3 signaling axis as a critical driver of MuSC death and inflammaging in PMD, and suggest that its targeted inhibition may offer a promising therapeutic strategy for preventing or treating PMD.

### The diagnostic value of the key functional hub DEGs in PMD patients

**Fig. 8:**
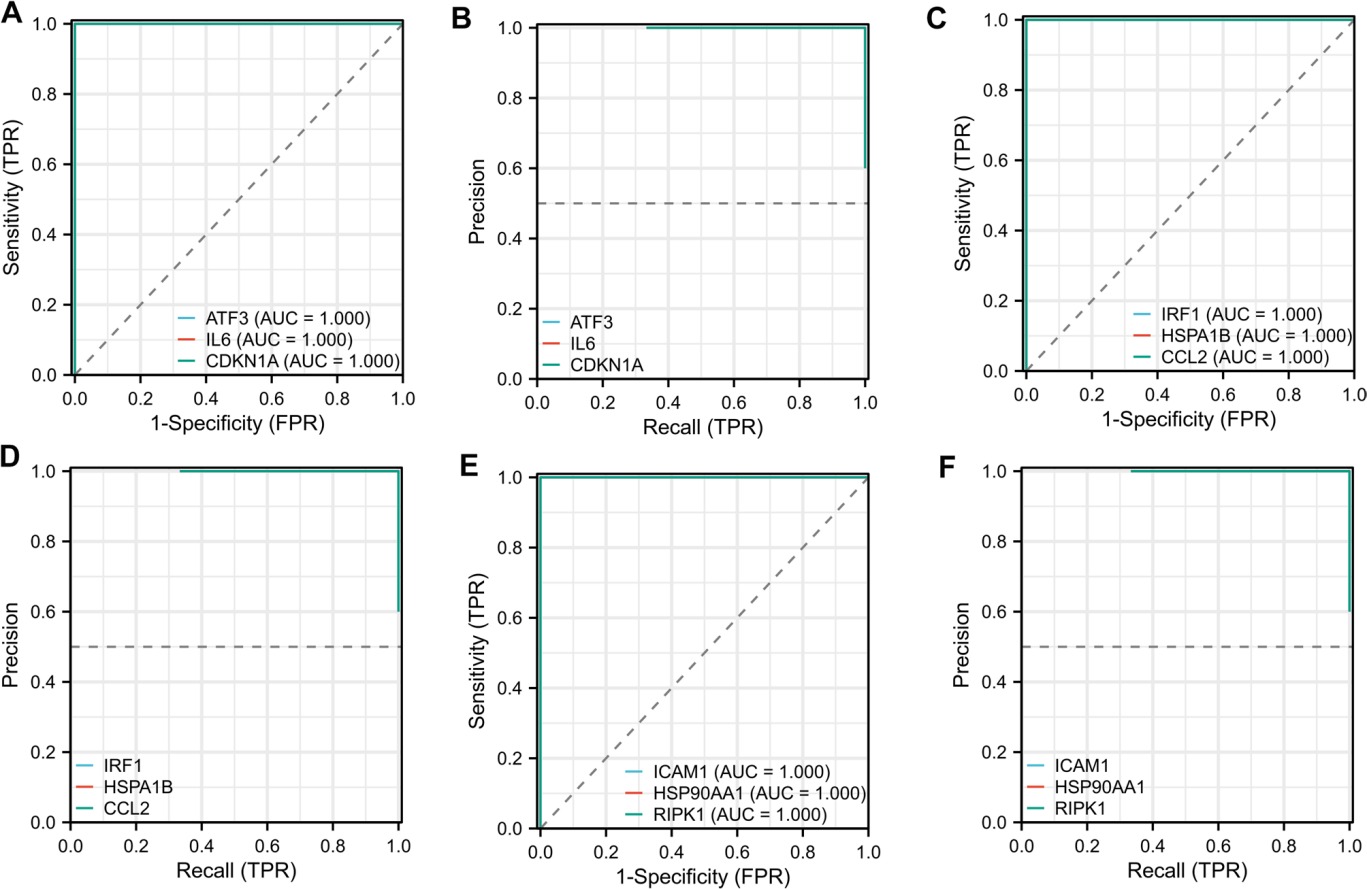
Evaluate the diagnostic value of key funtional hub DEGs in PMD. **A, C, E**: ROC curve analysis. The horizontal axis represents 1-specificity, and the closer the value is to zero, the higher the accuracy. The vertical axis represents sensitivity; the larger the value, the better the accuracy. **B, D, F**: PR curve analysis. The X-axis represents the recall rate, also known as the true positive rate. The closer the X-axis is to 1, the higher the accuracy; The Y-axis of the vertical axis is called accuracy, and the larger the Y-axis, the better the accuracy

The ROC curve illustrates the relationship between sensitivity and specificity, while PR curve highlights the balance between precision and recall. Thus, to evaluate the diagnostic potential of the key DEGs, we conducted both ROC and PR curve analyses (Fig. 8). The results shown that all hub DEGs exhibited an AUC value of 1, indicating perfect discriminatory power within this exploratory cohort. Although these findings are highly promising, they require validation in larger, independent cohorts. The findings indicate that ATF3 shows promising diagnostic potential for PMD, suggesting its possible utility as a clinical biomarker for PMD diagnosis.

## Discussion

The paravertebral muscles are primarily composed of the multifidus, erector spinae, and psoas major [1, 5]. Research on these muscles has largely focused on morphological characteristics, such as cross-sectional area and fat infiltration, while the underlying pathological mechanisms of PMD remain poorly understood. Differential gene expression plays a critical role in the onset and progression of various human diseases by regulating complex genetic networks and cell signaling cascades. Numerous clinical and basic studies have demonstrated that altered differential gene expression can lead to both microscopic changes such as impaired muscle cell function and macroscopic changes, including fat infiltration, fibrosis, and reduced muscle cross-sectional area [8–11, 17, 18, 31, 35]. Notably, knockdown of NOD-like receptor family pyrin domain containing 3 (NLRP3) has been shown to protect against PMD and scoliosis by remarkably inhibiting pyroptosis, apoptosis, and IL-1β expression in paravertebral muscle cells [36]. Similarly, features of PMD such as reducedmuscle cross-sectional area and increased fibrosis have been observed in Tuberous Sclerosis Complex 1 (TSC1) knockout mouse models [37]. Our team demonstrated that PMD occurs in patients with lumbar disc herniation, which is related to the high levels of TNF-α, IL-6, and IL-1β in multifidus [21, 38]. We have also identified differential expression of genes related to cell death, oxidative stress, and inflammatory responses in PMD [15, 17, 34]. Luo et al. [39] reported that Terminal Nucleotidyltransferase 5 A (Tent5a) knockout disrupts myogenic differentiation and type I fiber formation, implicating it in fibrotic pathways in paravertebral muscles of adolescent idiopathic scoliosis patients. In the present study, we established the transcriptomic profile of PMD, identifying ATF3, CDKN1A/P21, and IL6 as key upregulated DEGs. These genes appear to regulate critical pathological processes such as ferroptosis, apoptosis, muscle cell differentiation, ECM metabolism, pyroptosis, and inflammaging, thereby advancing our understanding of PMD pathogenesis.

Regulation of gene expression is a complex biological process, involving multiple layers of control, with microRNAs (miRNAs) and TFs being key mediators [40]. TFs regulate gene transcription by binding to specific sequences in the promoter regions of target genes through their DNA-binding domains [41]. Importantly, both miRNAs and TFs often interact within gene regulatory networks to co-regulate gene expression [14, 19, 20, 35]. These networks play vital roles in numerous physiological and pathological processes and are critically involved in the development of various degenerative conditions, including intervertebral disc degeneration [14, 19, 20, 35], spinal deformities [36, 39], and muscle degeneration [15, 24, 34]. In this study, we identified 409 DEGs, 10 key functional hub DEGs, and 12 DEMs associated with PMD using RNA sequencing. Based on these findings, we constructed regulatory networks linking TFs and miRNAs to the hub DEGs (Supplementary Figure S3). Analysis of the TF-gene co-regulatory network analysis highlighted the EGR1-ATF3 signaling pathway, consistent with our previous single-cell transcriptomic data from paravertebral muscle tissue [17]. While this study identified several potentially interesting miRNA signatures, comprehensive analysis of miRNA-mediated EGR1-ATF3 signaling and clinical correlations would require substantially more experimental validation and larger cohorts. Future research will focus on unraveling the precise roles and regulatory mechanisms of individual key molecules within these networks to deepen our understanding of PMD pathogenesis.

.Cells, the fundamental units of life, maintain homeostasis through a range of regulated cell death pathways, including ferroptosis, apoptosis, pyroptosis, and senescence [42, 43]. These pathways can function independently or in coordination, influencing both physiological and pathological outcomes [42, 43]. In our previous research, we observed the potential coexistence of apoptotic, pyroptotic, and ferroptotic mechanisms during intervertebral disc degeneration [14, 44]. There is reciprocal interplay between inflammatory signaling and cell death mechanisms. Inflammatory cell death, including pyroptosis and ferroptosis, elevates ROS and activates NF-κB signaling, thereby amplifying IL-1β/IL-6/IL-18/TNF-α production. This cascade promotes inflammaging and ECM catabolism, ultimately driving degenerative pathology [5, 9]. IL-6 also functions as a myokine involved in regulating muscle homeostasis, and its dysregulation has been closely linked to sarcopenia in aging ndividuals [11]. Interestingly, a recent study published in Nature demonstrated that genetic inhibition of interleukin-11 (IL-11), a member of the IL-6 cytokine family, significantly extended health span in mouse models, highlighting the therapeutic potential of targeting this signaling axis in age-related degeneration [10]. Our study provides experimental validation that ATF3 is a direct downstream target of EGR1. Functional assays using both overexpression and knockdown approaches demonstrated that EGR1 and ATF3 jointly regulate several pathological processes in MuSCs, including cell pyroptosis, ferroptosis, apoptosis, ROS accumulation, inflammaging, and ECM degradation. Collectively, our findings suggest that EGR1-ATF3 signaling axis plays a central role in driving MuSC cell death and inflammaging, forming a self-reinforcing feedback loop that contributes to the progression of PMD. Furthermore, ATF3 shows strong diagnostic potential and may serve as a promising biomarker and therapeutic target for the diagnosis and treatment of PMD.

.However, this study has several limitations that should be acknowledged. Firstly,, the sample size of paravertebral muscle tissues analyzed was relatively small, which may limit the universality and reliability of the results. More samples need to be obtained to confirm the universality and reproducibility of our findings in the future. Notably, the observed AUC = 1 in our pilot cohort likely reflects sample size limitations rather than true diagnostic perfection. Larger validation studies are needed to establish robust accuracy estimates. On the other hand, while our in vitro findings provide compelling mechanistic evidence, we acknowledge that the absence of in vivo validation represents a study limitation. Future investigations employing appropriate animal models of PMD will be essential to confirm the physiological relevance of these observations. Lastly, although this study focused on key hub genes, we cannot exclude the potential contributions of other differentially expressed genes that were not functionally characterized. Further in-depth investigations will be required to explore their roles and biological significance in PMD.

## Conclusion

In this study, human paravertebral muscle tissues were collected and subjected to RNA sequencing, leading to the identification of 409 DEGs associated with PMD. Bioinformatics analysis further revealed 81 functional DEGs linked to key pathological processes, including ferroptosis, apoptosis, pyroptosis, senescence, autophagy, inflammation, ECM metabolism, and muscle cell differentiation, proliferation, and regeneration, all of which contribute to PMD progression. The top 10 hub DEGs were found to be regulated by multiple TFs and demonstrated strong diagnostic potential. Notably, this study is the first to reveal that EGR1–ATF3 signaling promotes cell death and inflammaging, playing a central role in PMD pathogenesis. These findings offer valuable insights into novel therapeutic targets and strategies for the treatment of PMD.

## Electronic supplementary material

Below is the link to the electronic supplementary material.

Supplementary Material 1: Supplementary Table S1. The extracellular matrix genes, oxidative stress genes, ferroptosis genes, autophagy genes, senescence genes, inflammasome genes, pyroptosis genes, and apoptosis genes.

Supplementary Material 2: Supplementary Table S2. The forward primers and reverse primers of each gene used in this study.

Supplementary Material 3: Supplementary Table S3. The differential expression genes and miRNAs identified based on RNA sequencing.

Supplementary Material 4: Supplementary Table S4. The 81 functional differential expression genes related to PMD.

Supplementary Material 5

## Data Availability

Data is provided within the manuscript or supplementary information files.
