# Supplementary material for: Targeting EGR1-ATF3 signaling mitigates paravertebral muscle degeneration by regulating cell death and inflammaging
Source: Biol Res. 2025 Jul 28;58:52. doi: 10.1186/s40659-025-00634-1 (PMC12302741; doi:10.1186/s40659-025-00634-1)
Supplement: Supplementary file 1 — Supplementary Material 1: Supplementary Table S1. The extracellular matrix genes, oxidative stress genes, ferroptosis genes, autophagy genes, senescence genes, inflammasome genes, pyroptosis genes, and apoptosis genes. [file 40659_2025_634_MOESM1_ESM.pdf]

Autophagy	Senescence	Inflammasome	Pyroptosis	Ferroptosis
ATG12	ACD	CARD6	CASP6	AKR1C2
ATG3	AGO1	CARD19	CASP9	AKR1C3
ATG4A	AGO3	ARPC4	GPX4	RB1
ATG4B	AGO4	PYCARD	GSDMC	HSF1
ATG4C	ANAPC1	CARD10	IL6	GCLC
ATG4D	ANAPC10	NLRP2	NLRP3	SQSTM1
ATG5	ANAPC11	CARD14	NLRP6	NQO1
ATG7	ANAPC15	AIM2	NOD1	MUC1
BECN1	ANAPC16	CARD9	NOD2	MT1G
BECN2	ANAPC2	CARD11	PJVK	CISD1
GABARAP	ANAPC4	CASP1	PLCG1	FANCD2
GABARAPL1	ANAPC5	LRRC58	PRKACA	FTMT
GABARAPL2	ANAPC7	CARD6	PYCARD	HSPA5
IFNA14	ASF1A	LRRC39	SCAF11	HELLS
IFNA16	ATM	LRRC14B	TIRAP	SCD
IFNA4	BMI1	LRRC1	TNF	SRC
IFNA6	CABIN1	LRRC42		STAT3
IFNA13	CBX2	CARD19		PML
IFNG	CBX4	LRRC74A		MTOR
PIK3C3	CBX6	LRRC6		NFS1
PIK3R4	CBX8	LRRC74B		TP63
PRKAA1	CCNA1	LRRC23		CDKN1A
PRKAA2	CCNA2	LRRC30		MIR137
ULK1	CCNE1	LRRC8D		ENPP2
ULK2	CCNE2	LRRC66		FH
ULK3	CDC16	LRRC8C		CISD2
AMBRA1	CDC23	LRRC3C		MIR9-1
ARL13B	CDC26	LRRC8B		MIR9-2
ATG10	CDC27	LRRC4B		MIR9-3
ATG101	CDK2	LRRC71		ISCU
ATG12	CDK4	LRRC61		ACSL3
ATG13	CDK6	LRRC43		OTUB1
ATG14	CDKN1A	LRRC40		CD44
ATG16L1	CDKN1B	LRRC7		LINC00336
ATG3	CDKN2A	LRRC10B		BRD4
ATG4A	CDKN2B	LRRC9		PRDX6
ATG4B	CDKN2C	LRRC28		MIR17
ATG4C	CDKN2D	LRRC8E		NF2
ATG4D	CEBPB	LRRC18		ARNTL
ATG5	E2F1	NLRC4		JUN
ATG7	E2F2	LRRC37A		CA9
ATG9A	E2F3	LRRC36		TMBIM4
ATG9B	EED	LRRC57		PLIN2
ATM	EHMT1	LRRC75A		MIR212
BECN1	EHMT2	LRRC25		Fer1HCH
CETN1	EP400	LRRC14		AIFM2

CFTR	ERF	LGR5	LAMP2
CHMP2A	ETS1	LRRC73	ZFP36
CHMP2B	ETS2	LRRC24	PROM2
CHMP3	EZH2	LRRC45	CHMP5
CHMP4B	FOS	LRRC8A	CHMP6
CHMP4C	FZR1	ARPC4	CAV1
CHMP6	H1-0	LRRC31	GCH1
CHMP7	H1-1	LRRC47	DAZAP1
CSNK2A1	H1-2	LRRC4	PIR
CSNK2A2	H1-3	PYCARD	HCAR1
CSNK2B	H1-4	LRRC2	SLC16A1
DYNC1H1	H1-5	LGR6	NR4A1
DYNC1I1	H2AB2	LRRC3B	RPTOR
DYNC1I2	H2AC19	LRRC49	SREBF1
DYNC1LI1	H2AC18	LRRC19	SREBF2
DYNC1LI2	H2AC20	LRRC38	FZD7
DYNLL1	H2AC4	LRRC52	P4HB
DYNLL2	H2AJ	LRRC20	NT5DC2
EEF1A1	H2AC13	LRRC34	BCAT2
EPAS1	H2AX	LRRC3	PLA2G6
FUNDC1	H2AZ1	CARD10	MIR424
GABARAP	H2AZ2	NOD1	PARK7
GABARAPL1	H2BC1	LRRC15	FXN
GABARAPL2	H2BC4	LRRC63	SUV39H1
GFAP	H2BC21	LRRC41	ATF2
HDAC6	H2BC12	LRRC32	ACOT1
HSF1	H2BC7	NOD2	ALDH3A2
HSP90AA1	H2BC15	LRRC17	STK11
HSP90AB1	H2BC3	LRRC10	FNDC5
HSPA8	H2BC11	CARD14	CircIL4R
IFT88	H2BC13	LRRC55	CDH1
LAMP2	H2BC14	LRRC46	NEDD4L
LAMTOR1	H2BC18	LRRC59	BRD2
LAMTOR2	H2BC6	LGR4	BRD3
LAMTOR3	H2BC8	LRRC69	BRDT
LAMTOR4	H3-3B	LRRC26	DECR1
LAMTOR5	H3-3B	LRRC56	GLRX5
MAP1LC3A	H3C1	CARD9	NCOA3
MAP1LC3B	H3C8	LRRC27	NR5A2
MFN1	H3C10	CARD11	PANX2
MFN2	H3C11	LRRC4C	RHEBP1
MLST8	H3C6	CASP1	TFAP2A
MTERF3	H3C13	LRRC58	CP
MTMR14	H3C15	CARD6	ARF6
MTMR3	H3C3	LRRC39	ABHD12
MTOR	H3C2	LRRC14B	PPP1R13L
MVB12A	H3C7	LRRC1	TFAM

MVB12B	H4C4	LRRC42	KDM3B
NBR1	H4C1	CARD19	RNF113A
PARK7	H4C6	LRRC74A	AHCY
PCNT	H4C11	LRRC6	circ-TTBK2
PEX5	H4C2	LRRC74B	MIR522
PGAM5	H4C3	LRRC23	IDH2
PIK3C3	H4C14	LRRC30	PPARA
PIK3R4	H4C12	LRRC8D	SIAH2
PINK1	H4C8	LRRC66	VDR
PLIN2	H4C9	LRRC8C	NEDD4
PLIN3	HIRA	LRRC3C	AR
PRKAA1	HMGA1	LRRC8B	MTF1
PRKAA2	HMGA2	LRRC4B	COPZ1
PRKAB1	ID1	LRRC71	NUPR1
PRKAB2	IFNB1	LRRC61	USP35
PRKAG1	IGFBP7	LRRC43	NEAT1
PRKAG2	IL1A	LRRC40	PARP1
PRKAG3	IL6	LRRC7	PARP2
PRKN	JUN	LRRC10B	PARP3
RB1CC1	KAT5	LRRC9	PARP4
RHEB	KDM6B	LRRC28	PARP6
RNASE1	LMNB1	LRRC8E	PARP8
RPS27A	MAP2K3	LRRC18	PARP9
RPTOR	MAP2K4	LRRC37A	PARP10
RRAGA	MAP2K6	LRRC36	PARP11
RRAGB	MAP2K7	LRRC57	PARP12
RRAGC	MAP3K5	LRRC75A	PARP14
RRAGD	MAP4K4	LRRC25	PARP15
SLC38A9	MAPK1	LRRC14	PARP16
SQSTM1	DUSP16	LGR5	PDSS2
SRC	MAPK11	LRRC73	TXN
TOMM20	MAPK14	LRRC24	SENP1
TOMM22	MAPK3	LRRC45	OIP5-AS1
TOMM40	MAPK7	LRRC8A	MIR190A
TOMM5	MAPK8	ARPC4	FGF21
TOMM6	MAPK9	LRRC31	CREB1
TOMM7	MAPKAPK2	LRRC47	CREB3
TOMM70	MAPKAPK3	LRRC4	CREB5
TSC1	MAPKAPK5	LRRC2	MIR130B
TSC2	MDM2	LGR6	BEX1
TSG101	MDM4	LRRC3B	ASAH2
TUBA1A	MINK1	LRRC49	FABP4
TUBA1B	MOV10	LRRC19	AKT1S1
TUBA1C	MRE11	LRRC38	MLST8
TUBA4A	NBN	LRRC52	TYRO3
TUBA8	NFKB1	LRRC20	SIRT6
TUBAL3	PHC1	LRRC34	TMSB4X

TUBB1	PHC2	LRRC3	TMSB4Y
TUBB2A	PHC3	CARD10	KIF20A
TUBB2B	RAD50	NOD1	ECH1
TUBB3	RB1	NLRP2	circRHOT1
TUBB4A	RBBP4	LRRC15	ETV4
TUBB4B	RBBP7	LRRC63	MEG8
TUBB6	RELA	LRRC41	VCP
UBA52	RING1	LRRC32	circ_0007142
UBAP1	RNF2	NOD2	RBMS1
UBB	RPS27A	LRRC17	KDM4A
UBC	RPS6KA1	LRRC10	MGST1
UBE2N	RPS6KA2	CARD14	circKIF4A
UBE2V1	RPS6KA3	LRRC55	miR-7-5p
ULK1	SCMH1	LRRC46	circ_0067934
USP30	SP1	LRRC59	MPC1
UVRAG	STAT3	LGR4	CHMP1A
VCP	SUZ12	LRRC69	CAMKK2
VDAC1	TERF1	LRRC26	SOX2
VIM	TERF2	LRRC56	SRSF9
VPS28	TERF2IP	CARD9	PROK2
VPS37A	TFDP1	LRRC27	MIR4443
VPS37B	TFDP2	CARD11	SIRT2
VPS37C	TINF2	LRRC4C	circRNA1615
VPS37D	TNIK	CASP1	MIR27A
WDR45	TNRC6A	LRRC58	MIR670
WDR45B	TNRC6B	CARD6	MEF2C
WIPI1	TNRC6C	LRRC39	EZH2
WIPI2	TP53	LRRC14B	PEDS1
ARL13B	UBA52	LRRC1	ADAMTS13
CETN1	UBB	LRRC42	CDC25A
CFTR	UBC	CARD19	SFRS9
EEF1A1	UBE2C	LRRC74A	CAV
GFAP	UBE2D1	LRRC6	CircFNDC3B
HDAC6	UBE2E1	LRRC74B	PPARD
HSP90AA1	UBE2S	NLRP3	ENO3
HSP90AB1	UBN1	LRRC23	LCN2
HSPA8	ACD	LRRC30	MARCHF5
IFT88	ASF1A	LRRC8D	TRIB2
LAMP2	ATM	LRRC66	DHODH
PARK7	CABIN1	LRRC8C	MIR545
PCNT	CCNA1	LRRC3C	PDK4
PLIN2	CCNA2	LRRC8B	CircPVT1
PLIN3	CCNE1	LRRC4B	MIR9-3HG
RNASE1	CCNE2	LRRC71	ADIPOQ
RPS27A	CDK2	LRRC61	circDTL
UBA52	CDKN1A	LRRC43	mmu_circRNA_0000309
UBB	CDKN1B	LRRC40	PTPN18

UBC	EP400	LRRC7	ABCC5
VIM	H1-0	LRRC10B	CISD3
ARL13B	H1-1	LRRC9	MS4A15
CETN1	H1-2	LRRC28	FURIN
CFTR	H1-3	LRRC8E	circRHBG
CHMP2A	H1-4	LRRC18	GALNT14
CHMP2B	H1-5	LRRC37A	KLHDC3
CHMP3	H2AB2	LRRC36	LINC01833
CHMP4B	H2AC19	LRRC57	circGFRA1
CHMP4C	H2AC18	LRRC75A	MAPKAP1
CHMP6	H2AC20	LRRC25	PRR5
CHMP7	H2AC4	LRRC14	RICTOR
HDAC6	H2AJ	LGR5	GSTM1
HSPA8	H2AC13	LRRC73	TERT
IFT88	H2AX	LRRC24	circ0097009
MVB12A	H2AZ1	LRRC45	TMEM161B-DT
MVB12B	H2AZ2	LRRC8A	circEPSTI1
PARK7	H2BC1	ARPC4	MIR18A
PCNT	H2BC4	LRRC31	RARRES2
PLIN2	H2BC21	LRRC47	HSPB1
PLIN3	H2BC12	LRRC4	SLC40A1
RNASE1	H2BC7	PYCARD	GPX4
RPS27A	H2BC15	LRRC2	NFE2L2
TSG101	H2BC3	LGR6	FTH1
UBA52	H2BC11	LRRC3B	SLC3A2
UBAP1	H2BC13	LRRC49	CBS
UBB	H2BC14	LRRC19	SESN2
UBC	H2BC18	LRRC38	FTL
VIM	H2BC6	LRRC52	RRM2
VPS28	H2BC8	LRRC20	GDF15
VPS37A	H4C4	LRRC34	NOS2
VPS37B	H4C1	LRRC3	RELA
VPS37C	H4C6	CARD10	PRDX1
VPS37D	H4C11	NOD1	DUSP1
BAK1	H4C2	NLRP2	NCF2
BAX	H4C3	LRRC15	MT3
CASP1	H4C14	LRRC63	UBC
CASP3	H4C12	LRRC41	ALB
CASP4	H4C8	LRRC32	TXNRD1
CHMP2A	H4C9	NOD2	SRXN1
CHMP2B	HIRA	LRRC17	GPX2
CHMP3	HMGA1	LRRC10	BNIP3
CHMP4B	HMGA2	CARD14	OXSR1
CHMP4C	KAT5	LRRC55	SELENOS
CHMP6	LMNB1	LRRC46	ANGPTL7
CHMP7	MRE11	LRRC59	DDIT4
CYCS	NBN	LGR4	LOC284561

ELANE	RAD50	LRRC69	ASNS
GSDMD	RB1	LRRC26	TSC22D3
GSDME	TERF1	LRRC56	DDIT3
GZMB	TERF2	CARD9	JDP2
HMGB1	TERF2IP	LRRC27	SLC1A4
IL18	TINF2	CARD11	PCK2
IL1A	TP53	LRRC4C	TXNIP
IL1B	UBN1	CASP1	VLDLR
IRF1	ASF1A	NLRP3	GPT2
IRF2	CABIN1	NLRP14	PSAT1
TP53	CDKN1A	NLRC3	LURAP1L
ARL13B	EP400	NLRC4	SLC7A5
ATG12	H1-0	NLRP10	HERPUD1
ATG5	H1-1	NLRP5	XBP1
ATM	H1-2	NOD1	ZNF419
CETN1	H1-3	NLRC5	KLHL24
CFTR	H1-4	NOD2	TRIB3
CSNK2A1	H1-5	CIITA	ZFP69B
CSNK2A2	HIRA	NLRX1	ATP6V1G2
CSNK2B	HMGA1	NLRP12	VEGFA
DYNC1H1	HMGA2	NLRP6	TUBE1
DYNC1I1	LMNB1	CASP8	ARRDC3
DYNC1I2	RB1	IL1B	CEBPG
DYNC1LI1	TP53	MALT1	SNORA16A
DYNC1LI2	UBN1	NFKB1	RGS4
DYNLL1	AGO1	PYCARD	BLOC1S5-TXNDC5
DYNLL2	AGO3	RELA	LOC390705
EPAS1	AGO4	AIM2	EIF2S1
FUNDC1	CDK4	APP	KIM-1
HDAC6	CDK6	BCL2	CXCL2
HSF1	CDKN2A	BCL2L1	HSD17B11
HSP90AA1	CDKN2B	CASP1	SETD1B
HSPA8	CDKN2C	HMOX1	MAFG
IFT88	CDKN2D	HSP90AB1	IL33
MAP1LC3A	E2F1	MEFV	HAMP
MAP1LC3B	E2F2	NFKB1	STEAP3
MFN1	E2F3	NFKB2	DRD5
MFN2	ERF	NLRC4	DRD4
MTERF3	ETS1	NLRP3	MAP3K5
NBR1	ETS2	P2RX7	SLC2A1
PARK7	ID1	PANX1	SLC2A3
PCNT	MAPK1	PSTPIP1	SLC2A6
PEX5	MAPK3	PYCARD	SLC2A8
PGAM5	MDM2	RELA	SLC2A12
PINK1	MDM4	SUGT1	GLUT13
PLIN2	MOV10	TXNIP	SLC2A14
PLIN3	RB1	APP	EIF2AK4

PRKAA2	RPS27A	CASP1	TFAP2C
PRKAB1	SP1	HMOX1	SP1
PRKAB2	TFDP1	HSP90AB1	HBA1
PRKAG1	TFDP2	MEFV	NNMT
PRKAG2	TNRC6A	NFKB1	PLIN4
PRKAG3	TNRC6B	NFKB2	HIC1
PRKN	TNRC6C	NLRP3	STMN1
RPS27A	TP53	P2RX7	CAPG
SQSTM1	UBA52	PANX1	HNF4A
SRC	UBB	PSTPIP1	NGB
TOMM20	UBC	PYCARD	YWHAE
TOMM22	AGO1	RELA	GABPB1
TOMM40	AGO3	SUGT1	AURKA
TOMM5	AGO4	TXNIP	MIR4715
TOMM6	BMI1	CASP1	RIPK1
TOMM7	CBX2	IL1B	MIR30B
TOMM70	CBX4	NFKB1	MMP13
TUBA1A	CBX6	NLRP3	LRRFIP1
TUBA1B	CBX8	PYCARD	CBR1
TUBA1C	CDK4	RELA	PTGS2
TUBA4A	CDK6	TRAF3	AKR1C1
TUBA8	CDKN2A	CASP1	RPL8
TUBAL3	CDKN2B	CTSB	IREB2
TUBB1	CDKN2C	IL1B	ATP5MC3
TUBB2A	CDKN2D	NFKB1	CS
TUBB2B	E2F1	NOX1	EMC2
TUBB3	E2F2	PYCARD	ACSF2
TUBB4A	E2F3	TLR4	NOX1
TUBB4B	EED	CPTP	CYBB
TUBB6	EZH2	MEFV	NOX3
UBA52	FOS	NLRC3	NOX4
UBB	H2AB2	SIRT2	NOX5
UBC	H2AC19	AIM2	DUOX1
UBE2N	H2AC18	ATAT1	DUOX2
UBE2V1	H2AC20	CD36	G6PD
ULK1	H2AC4	CPTP	PGD
USP30	H2AJ	DDX3X	VDAC2
VCP	H2AC13	DHX33	PIK3CA
VDAC1	H2AX	EIF2AK2	FLT3
VIM	H2AZ1	GBP5	SCP2
AKT1S1	H2AZ2	MEFV	TP53
DEPTOR	H2BC1	NLRC3	ACSL4
ATG10	H2BC4	SIRT2	LPCAT3
ATG12	H2BC21	TLR4	NRAS
ATG13	H2BC12	TLR6	KRAS
ATG16L1	H2BC7	USP50	HRAS
ATG16L2	H2BC15	ATAT1	TF

ATG3	H2BC3	CD36	TFRC
ATG4A	H2BC11	DDX3X	TFR2
ATG5	H2BC13	DHX33	SLC38A1
ATG7	H2BC14	GBP5	SLC1A5
BECN1	H2BC18	TLR4	GLS2
MAP1LC3A	H2BC6	TLR6	GOT1
MTOR	H2BC8	USP50	CARS1
PIK3C3	H3-3B	AIM2	ALOX5
PIK3R4	H3-3B	CASP1	KEAP1
RB1CC1	H3C1	CASP4	HMOX1
ULK1	H3C8	PYCARD	ATG5
ULK2	H3C10	AIM2	ATG7
WIPI1	H3C11	CASP1	NCOA4
ZFYVE1	H3C6	CASP4	ALOX12
AKT1S1	H3C13	DDX3X	ALOX12B
AMBRA1	H3C15	DHX33	ALOX15
ATG10	H3C3	GSDMD	ALOX15B
ATG12	H3C2	NLRC4	ALOXE3
ATG13	H3C7	NLRP3	PHKG2
ATG14	H4C4	NLRP6	ACO1
ATG16L1	H4C1	PYCARD	G6PDX
ATG3	H4C6	CASP1	ULK1
ATG5	H4C11	CASP4	ATG3
ATG7	H4C2	NLRC4	ATG4D
BCL2	H4C3	CASP1	BECN1
BECN1	H4C14	CASP4	MAP1LC3A
BMI1	H4C12	DDX3X	GABARAPL2
BMP2	H4C8	DHX33	GABARAPL1
BRAF	H4C9	GSDMD	ATG16L1
CCL3	IFNB1	NLRP3	WIPI1
CD44	JUN	PYCARD	WIPI2
CDC25B	KDM6B		SNX4
CDKN1A	MAP2K3		ATG13
CDKN1B	MAP2K4		ULK2
CDKN2A	MAP2K6		SAT1
CEBPB	MAP2K7		EGFR
COL10A1	MAP3K5		MAPK3
COL1A1	MAP4K4		MAPK1
COL3A1	MAPK1		BID
CREG1	DUSP16		ZEB1
CXCL1	MAPK11		DPP4
CXCL2	MAPK14		CDKN2A
CXCL3	MAPK3		PEBP1
CXCL14	MAPK8		SOCS1
E2F1	MAPK9		CDO1
FKBP8	MAPKAPK2		MYB
FN1	MAPKAPK3		MAPK8

GABARAP	MAPKAPK5	MAPK9
GABARAPL1	MDM2	CHAC1
GABARAPL2	MDM4	MAPK14
GSK3B	MINK1	LINC00472
GSN	MOV10	PRKAA2
HMGA1	PHC1	PRKAA1
HRAS	PHC2	ELAVL1
IFNB1	PHC3	BAP1
IFNG	RBBP4	ABCC1
IGF1	RBBP7	MIR6852
IGF1R	RING1	ACVR1B
IGFBP3	RNF2	TGFBR1
IGFBP5	RPS27A	EPAS1
IGFBP7	SCMH1	HILPDA
IL1A	SUZ12	HIF1A
IL1B	TFDP1	IFNG
IL24	TFDP2	ANO6
IL3	TNIK	LPIN1
IL6	TNRC6A	HMGB1
IL6R	TNRC6B	TNFAIP3
IL6ST	TNRC6C	TLR4
ING1	TP53	ATF3
ING2	UBA52	ATM
INHBA	UBB	YY1AP1
IRF1	UBC	EGLN2
IRF5	ANAPC1	MIOX
IRF7	ANAPC10	TAZ
JUN	ANAPC11	MTDH
KMT2A	ANAPC15	IDH1
LAMP1	ANAPC16	SIRT1
LAMP2	ANAPC2	FBXW7
MAP1LC3A	ANAPC4	PANX1
MAP1LC3B	ANAPC5	DNAJB6
MAP2K1	ANAPC7	BACH1
MAP2K3	CCNA1	LONP1
MAPK1	CCNA2	CD82
MAPK14	CDC16	IL1B
MDM2	CDC23	CTSB
MLST8	CDC26	POR
MMP14	CDC27	CYB5R1
MTOR	CDK2	ELOVL5
PCNA	CDK4	FADS1
PIK3C3	CDK6	FBW7
PLAT	CDKN1A	PTEN
PLAU	CDKN1B	NR1D1
PTEN	CDKN2A	NR1D2
RAF1	CDKN2B	TBK1

RB1	CDKN2C	IL6
RB1CC1	CDKN2D	USP7
RNASEL	CEBPB	miR-182-5p
RSL1D1	EHMT1	miR-378a-3p
SERPINB2	EHMT2	ATF4
SERPINE1	FOS	AQP3
SH3GLB1	FZR1	AQP5
SLC39A1	H2AB2	AQP8
SLC39A2	H2AC19	LINC00618
SLC39A3	H2AC18	MT1DP
SLC39A4	H2AC20	PEX10
SMAD3	H2AC4	AGPAT3
SMAD4	H2AJ	PEX12
SPARC	H2AC13	CHP1
SQSTM1	H2AX	GPAT4
SRC	H2AZ1	BRPF1
TGFB1	H2AZ2	OSBPL9
THBS1	H2BC1	INTS2
TNFSF15	H2BC4	MMD
TP53	H2BC21	CYP4F8
ULK1	H2BC12	MLLT1
UVRAG	H2BC7	TTPA
VTN	H2BC15	GRIA3
AMBRA1	H2BC3	EPT1
ATG12	H2BC11	POM121L12
ATG13	H2BC13	LIG3
ATG14	H2BC14	AEBP2
ATG2A	H2BC18	AGPS
ATG2B	H2BC6	CDCA3
ATG3	H2BC8	PEX2
ATG5	H3-3B	PEX6
ATG7	H3-3B	TIMM9
ATG9A	H3C1	DCAF7
ATG9B	H3C8	LCE2C
ATP13A2	H3C10	FAR1
ATP5IF1	H3C11	PHF21A
BECN1	H3C6	SMAD7
BNIP3	H3C13	LYRM1
BNIP3L	H3C15	AMN
CAMKK2	H3C3	PEX3
CDC37	H3C2	MTCH1
CISD2	H3C7	ACADSB
CSNK2A2	H4C4	PVT1
CTSK	H4C1	hsa_circ_0008367
CTTN	H4C6	SLC39A14
DNM1L	H4C11	MAP3K11
FBXO7	H4C2	GSK3B

FBXW7	H4C3	BRD7
FIS1	H4C14	SLC25A28
FUNDC1	H4C12	MFN2
FUNDC2	H4C8	SLC11A2
FZD5	H4C9	ZFAS1
GABARAP	IGFBP7	TSC1
GABARAPL1	IL1A	TGFB1
GABARAPL2	IL6	SNCA
GBA	JUN	SIRT3
GSK3A	MAPK1	CGAS
HAX1	MAPK3	STING1
HDAC6	MAPK7	HDDC3
HIF1A	NFKB1	MIR761
HK2	RELA	MDM2
HTRA2	RPS27A	MDM4
HTT	RPS6KA1	MIR214
HUWE1	RPS6KA2	DLD
MAP1LC3A	RPS6KA3	WWTR1
MAP1LC3B	STAT3	PRKCA
MARK2	UBA52	LGMN
MFN2	UBB	SMPD1
MUL1	UBC	MYCN
OPTN	UBE2C	IFNA1
PARK7	UBE2D1	IFNA2
PHB2	UBE2E1	IFNA4
PINK1	UBE2S	IFNA5
PPARGC1A	ALDOC	IFNA6
PRKN	ENO1	IFNA7
RB1CC1	GAPDH	IFNA8
RNF41	HK1	IFNA10
SPATA18	LDHA	IFNA13
SQSTM1	PGK1	IFNA14
SREBF1	PKM	IFNA16
SREBF2	PRKAA1	IFNA17
TIGAR	RB1	IFNA21
TOMM7	TP53	SMG9
TP53	AADAT	PPARG
TSC2	ACMSD	miR-335
TSPO	AFMID	SNX5
USP30	AHR	PAQR3
USP36	CDKN1A	MICU1
VDAC1	EIF2AK1	TOR2A
VPS13C	EIF2AK4	MIR375
VPS13D	FOXO1	MAP3K14
WDR45	HAAO	CircKDM4C
WDR45B	IDO1	MIR324
WIPI1	IDO2	QSOX1

WIPI2	IFNB1	MIB2
ATG12	IFNG	CLTRN
ATG13	IL1R2	KLF2
ATG2A	KLF5	MIR5096
ATG2B	KMO	HOTAIR
ATG3	KYNU	H19
ATG5	NOS1	FOXO4
ATG7	QPRT	YTHDC2
ATG9A	TDO2	DDR2
ATG9B	TLR4	SLC39A7
RB1CC1	TNF	TRIM46
WDR45	TP53	ACSL1
WDR45B	ELAVL1	KDM5A
WIPI1	GOT1	TRIM21
WIPI2	GOT2	DPEP1
ACBD5	HMGA1	CYGB
PIK3C3	IL1B	IDO1
PIK3R4	IL6	GSTZ1
PJVK	MDH2	GJA1
RB1CC1	NAMPT	SLC7A11
TRAPPC8	NMNAT2	PGRMC1
ATG7	PARP1	CIRBP
ATP13A2	PRKAA1	circPSEN1
BAG3	RB1	USP11
CLU	RELA	YAP
CTSA	SCO2	MIR135B
EEF1A2	SIRT1	TRIM26
PLK3	SIRT2	NDRG1
SNCA	SIRT3	MIR302A
SNRNP70	SIRT5	ASMTL-AS1
STUB1	SLC2A1	FADS2
SYNPO2	SLC2A4	PIEZO1
ATG13	TP53	LIFR
ATG2A	BRAF	PTPN6
ATG2B	FLNB	MIR15A
ATG7	GOT1	EGR1
RB1CC1	ME1	ADAM23
ADPRH	ME2	ARHGEF26-AS1
ADRB2	PDHA1	CPEB1
AKT1	PDK1	COX4I2
AMBRA1	PDP2	lncRNA AABR07017145.[illegible]
ATG10	TP53	TIMP1
ATG101	ABL1	KDM6B
ATG12	AKT3	METTL14
ATG13	ARG2	MIB1
ATG14	ARNTL	KDM5C
ATG16L1	B2M	MEG3

ATG16L2	BCL2L12	CCDC6
ATG2A	BCL6	CFL1
ATG2B	BMPR1A	MIR539
ATG3	CALR	KMT2D
ATG4B	CGAS	
ATG5	ECRG4	
ATG7	EEF1E1	
ATG9A	FBXO5	
ATG9B	HRAS	
ATP13A2	ID2	
ATP6V0A1	ING2	
ATP6V0A2	KAT6A	
ATP6V0B	KIR3DL2	
ATP6V0C	KRAS	
ATP6V0D1	MAP2K1	
ATP6V0D2	MAP3K3	
ATP6V0E1	NEK4	
ATP6V0E2	NEK6	
ATP6V1A	NSMCE2	
ATP6V1B1	NUAK1	
ATP6V1B2	OPA1	
ATP6V1C1	PAWR	
ATP6V1C2	PLA2R1	
ATP6V1D	PLK2	
ATP6V1E1	PML	
ATP6V1E2	PNPT1	
ATP6V1G1	PRKCD	
ATP6V1G2	PRKDC	
ATP6V1H	PRMT6	
AUP1	RBL1	
BAG3	RSL1D1	
BECN1	SIRT1	
BECN2	SLC30A10	
BNIP3	SMC5	
BNIP3L	SMC6	
C9orf72	SPI1	
CALCOCO2	SRF	
CAPN1	TBX2	
CAPNS1	TBX3	
CASP3	TERT	
CDC37	TWIST1	
CDK5	ULK3	
CDK5R1	VASH1	
CHMP2A	WNT16	
CHMP2B	YBX1	
CHMP3	YPEL3	
CHMP4B	ZKSCAN3	

CHMP4C	ZMPSTE24
CHMP6	ABL1
CLEC16A	AKT3
CLN3	BCL2L12
CSNK2A1	BCL6
CSNK2A2	CDK6
CSNK2B	FBXO5
DCN	HMGA2
DDRGK1	MAP3K3
DYNLL1	PLK2
DYNLL2	PRKDC
EI24	RBL1
KIAA1324	SIRT1
EMC6	SLC30A10
EPG5	TERF2
EPM2A	TERT
ERN1	TWIST1
EXOC1	YBX1
EXOC4	ZKSCAN3
EXOC7	ATM
EXOC8	ATR
FEZ1	CDKN1A
FEZ2	CDKN2A
FUNDC1	CHEK1
FYCO1	CHEK2
GABARAP	CTC1
GABARAPL1	ERCC1
GABARAPL2	MME
GAPDH	PLA2R1
GBA	ROMO1
GNAI3	SERPINE1
GPSM1	TERT
HDAC10	TP53
HDAC6	WNT16
HGS	WRN
HIF1A	ARNTL
HMOX1	CDKN1A
HSPB8	MAPK14
HTRA2	MAPKAPK5
HTT	PLA2R1
HUWE1	SIRT1
IFT20	TP53
IFT88	WNT16
IKBKG	MIF
IL4	CXCL11
ILRUN	NGF
IRGM	MDM2

KDR	TGFB1
KLHL3	EZH2
LAMP2	HIRA
LAMTOR1	EED
LAMTOR2	SUZ12
LAMTOR3	CBX7
LAMTOR4	E2F1
LAMTOR5	ERCC1
LARP1	NFKB1
LGALS8	CCN1
LIX1	CABIN1
LIX1L	UBN1
LRRK2	PIGF
LRSAM1	FAS
LZTS1	CTSB
MAP1LC3A	IL15
MAP1LC3B	ANG
MAP3K7	VPS51
MAPK3	IGFBP4
MAPK8	IL6ST
MCOLN1	EGF
MFN1	CCL20
MFN2	FGF2
MFSD8	PLAT
MLST8	TNFRSF1A
MTERF3	IL7
MTM1	TNFRSF11B
MTMR14	IL37
MTMR3	CCL8
MTMR7	ANGPT4
MTOR	IGFBP6
MVB12A	COL18A1
NBR1	EREG
NEDD4	AREG
NOD1	IGFBP7
NOD2	TIMP2
NPC1	HGF
NPRL2	TNFRSF1B
NPRL3	MMP14
NRBP2	PLAUR
NSFL1C	PLAU
NUPR1	IL13
OPTN	MIF
PACS2	ICAM1
PAFAH1B2	CXCL12
PGAM5	CCL3
PHB2	MMP13

PHF23	IL1A
PIK3C2B	IGFBP2
PIK3C3	EGFR
PIK3CA	SERPINE1
PIK3R4	IGFBP3
PIKFYVE	MKI67
PINK1	FGF7
PIP4K2A	MMP12
PIP4K2B	SERPINB2
PIP4K2C	IL1B
PJVK	IL6
PLAA	MMP3
POLDIP2	IL15
PRKAA1	IFNG
PRKAA2	IGFBP4
PRKAB1	IL6ST
PRKAB2	EGF
PRKACA	FGF2
PRKAG1	IL7
PRKAG2	TNFRSF11B
PRKAG3	IL37
PRKN	CCL8
PSEN1	IGFBP6
QSOX1	IGFBP7
RAB19	CCL11
RAB1A	MMP14
RAB1B	PLAUR
RAB23	IL13
RAB3GAP1	CXCL12
RAB3GAP2	CCL3
RAB43	MMP13
RAB5A	IL1A
RAB7A	IGFBP2
RALB	IGFBP3
RB1CC1	FGF7
RETREG1	MMP12
RETREG3	IL1B
RHEB	IL6
RIPK2	MMP3
RNF41	PIGF
RNF5	FAS
RPTOR	CTSB
RRAGA	IL15
RRAGB	ANG
RRAGC	VPS51
RRAGD	IGFBP4
RUBCN	IL6ST

RUBCNL	EGF
RUFY4	CCL20
SCFD1	FGF2
SCOC	PLAT
SEC22B	TNFRSF1A
SESN1	CHEK2
SESN2	IL7
SESN3	TNFRSF11B
SH3GLB1	IL37
SIRT1	CCL8
SLC38A9	GLB1
SMCR8	ANGPT4
SMG1	IGFBP6
SMURF1	COL18A1
SNAP29	EREG
SNAPIN	AREG
SNF8	IGFBP7
SNX14	TIMP2
SNX32	HGF
SNX5	TNFRSF1B
SNX6	MMP14
SPTLC1	PLAUR
SPTLC2	PLAU
SQSTM1	IL13
SRC	CDKN1A
STAM	TP53
STAM2	MIF
STBD1	ICAM1
STING1	CXCL12
STX12	CCL3
STX17	NOTCH1
SYNPO2	MMP13
TBC1D12	IL1A
TBC1D14	IGFBP2
TBC1D25	EGFR
TBC1D5	SERPINE1
TBK1	DPP4
TCIRG1	IGFBP3
TECPR1	MKI67
TEX264	FGF7
TIGAR	MMP12
TMEM39A	SERPINB2
TMEM39B	IL1B
TMEM41B	IL6
TMEM74	MMP3
TOMM20	IL15
TOMM22	IFNG

TOMM40	IGFBP4
TOMM5	IL6ST
TOMM6	BCL2L1
TOMM7	EGF
TOMM70	FGF2
TP53	CHEK2
TRAPPC8	IL7
TRIM13	TNFRSF11B
TSC1	IL37
TSC2	CCL8
TSG101	IGFBP6
UBA5	IGFBP7
UBQLN1	CCL11
UBQLN2	MMP14
UBQLN4	BCL2L2
UBXN2A	PLAUR
UBXN2B	IL13
UBXN6	CDKN1A
UCHL1	FOXO4
UFC1	CXCL12
UFL1	BCL2
UFM1	CCL3
ULK1	NOTCH1
USP30	MMP13
USP36	IL1A
UVRAG	IGFBP2
VAMP8	DPP4
VCP	IGFBP3
VDAC1	FGF7
VMP1	MMP12
VPS13C	IL1B
VPS13D	IL6
VPS16	MMP3
VPS25	
VPS26A	
VPS26B	
VPS28	
VPS33A	
VPS35	
VPS36	
VPS37A	
VPS37B	
VPS37C	
VPS37D	
VPS41	
VPS4A	
VPS4B	

VTA1
WAC
WDFY3
WDR45
WDR45B
WDR81
WIPI1
WIPI2
YOD1
ZFYVE1
ATG5
ATG7
EIF2AK4
IRGM
PHB2
ULK1
ULK2
ADRA1A
AKT1
ATG7
BCL2
BECN1
BMF
CHMP4B
CLEC16A
CPTP
CTSA
DAP
DAPL1
EIF4G1
EIF4G2
FEZ1
FEZ2
FOXK1
FOXK2
GATA4
GOLGA2
HERC1
HGF
HMOX1
HTR2B
IL10
IL10RA
KDM4A
KLHL22
LEP
LEPR

LRRK2
LZTS1
MCL1
MET
MT3
MTM1
MTMR7
MTMR9
MTOR
NPC1
NRBP2
NUPR1
PHF23
PIK3CA
PINK1
POLDIP2
PTPN22
QSOX1
RASIP1
RNF41
RNF5
RRAGA
RUBCN
SCFD1
SEC22B
SIRT2
SMCR8
SMG1
SNCA
SNRNP70
STAT3
TAB2
TAB3
TBC1D14
TIGAR
TLK2
TMEM39A
TMEM39B
TP53
TREM2
TSC1
TSC2
TSPO
UBQLN4
USP30
USP36
WASHC1

WDR6
ZKSCAN3
PINK1
RNF41
TIGAR
TP53
TSC2
TSPO
USP30
AKT1
BECN1
CHMP4B
CLEC16A
FEZ1
FEZ2
HMOX1
LRRK2
LZTS1
MTM1
MTOR
NPC1
NRBP2
NUPR1
PHF23
PIK3CA
PINK1
POLDIP2
QSOX1
RNF41
RUBCN
SCFD1
SEC22B
SMCR8
SMG1
TIGAR
TMEM39A
TMEM39B
TP53
TSC1
TSC2
UBQLN4
USP30
USP36
ADRB2
AMBRA1
ATF6
BAD

BAG3
BCL2L11
BECN1
BNIP3
BNIP3L
C9orf72
CALCOCO2
CAMKK2
DAPK1
DCN
DHRSX
EIF2AK4
KIAA1324
EPM2A
FBXO7
FLCN
FOXO1
FOXO3
FYCO1
GNAI3
GPSM1
GSK3A
GSK3B
HIF1A
HMGB1
HMOX1
HSPB8
HTT
IFNG
IKBKG
IL4
IRGM
KAT5
KDR
LARP1
LRRK2
LRSAM1
MAP3K7
MAPK3
MEFV
MID2
MTDH
NOD1
NOD2
NPRL2
OPTN
ORMDL3

PAFAH1B2
PARK7
PHB2
PIK3C2A
PIK3CB
PIM2
PINK1
PIP4K2A
PIP4K2B
PIP4K2C
PLEKHF1
PLK2
PLK3
PRKAA1
PRKAA2
PRKD1
PRKN
RAB3GAP1
RAB3GAP2
RALB
RIPK2
RNF152
ROCK1
RUFY4
SCOC
SESN1
SESN2
SESN3
SH3BP4
SH3GLB1
SIRT1
SMCR8
SPTLC1
SPTLC2
STING1
STK11
SVIP
TBK1
TFEB
TICAM1
TMEM59
TPCN1
TRIM13
TRIM14
TRIM21
TRIM5
TRIM27

TRIM38
TRIM6
TRIM65
TRIM68
TRIM8
TRIML1
TRIML2
TSC1
TSC2
UFL1
ULK1
UVRAG
VDAC1
VPS13D
WAC
ZC3H12A
BNIP3
CAMKK2
FBXO7
HIF1A
HTT
PARK7
PRKN
VDAC1
VPS13D
ADRB2
BAG3
BECN1
BNIP3
BNIP3L
C9orf72
CALCOCO2
DCN
KIAA1324
EPM2A
FYCO1
GNAI3
GPSM1
HIF1A
HMOX1
HSPB8
HTT
IKBKG
IL4
IRGM
KDR
LARP1

LRSAM1
MAP3K7
MAPK3
NOD1
NOD2
OPTN
PAFAH1B2
PINK1
PIP4K2A
PIP4K2B
PIP4K2C
PRKAA2
RAB3GAP1
RAB3GAP2
RALB
RIPK2
RUFY4
SCOC
SESN1
SESN2
SESN3
SH3GLB1
SIRT1
SMCR8
SPTLC1
SPTLC2
STING1
TBK1
TRIM13
TSC1
TSC2
ULK1
UVRAG
VDAC1
VPS13D
WAC
AIM2
APIP
CASP8
DHX9
GSDMA
GZMA
NLRC4
ZBP1
ABL1
ABL2
ACER2

ADRA1A
ADRB2
AKT1
AMBRA1
ATF6
ATG101
ATG13
ATM
ATP13A2
ATP6V0A1
ATP6V0A2
ATP6V0B
ATP6V0C
ATP6V0D1
ATP6V0D2
ATP6V0E1
ATP6V0E2
ATP6V1A
ATP6V1B1
ATP6V1B2
ATP6V1C1
ATP6V1C2
ATP6V1D
ATP6V1E1
ATP6V1E2
ATP6V1G1
ATP6V1G2
ATP6V1H
BAD
BAG3
BCL2
BCL2L11
BMF
BNIP3
BNIP3L
BOK
C9orf72
CALCOCO2
CAMKK2
CAPN1
CAPNS1
CASP1
CASP3
CDK5
CDK5R1
CHMP4B
CISD2

CLEC16A
CLN3
CPTP
CSNK2A2
CTSA
CTTN
DAP
DAPK1
DAPK2
DAPK3
DAPL1
DCN
DDIT3
DEPDC5
DEPP1
DHRSX
DNM1L
DRAM1
DRAM2
EEF1A1
EEF1A2
EIF2AK4
EIF4G1
EIF4G2
KIAA1324
EP300
EPM2A
ERCC4
ERN1
EXOC1
EXOC4
EXOC7
EXOC8
FBXL2
FBXO7
FBXW7
FEZ1
FEZ2
FLCN
FOXK1
FOXK2
FOXO1
FOXO3
FYCO1
FZD5
GAPDH
GATA4

GBA
GFAP
GNAI3
GOLGA2
GPR137
GPR137B
GPSM1
GSK3A
GSK3B
HAX1
HDAC6
HERC1
HGF
HIF1A
HMGB1
HMOX1
HSPB1
HSPB8
HTR2B
HTRA2
HTT
IKBKG
IL10
IL10RA
IL4
IRGM
ITPR1
KAT5
KAT8
KDM4A
KDR
KEAP1
KLHL22
LAMP3
LAMTOR1
LAMTOR2
LAMTOR3
LAMTOR4
LAMTOR5
LARP1
LEP
LEPR
LRRK2
LRSAM1
LZTS1
MAP3K7
MAPK15

MAPK3
MAPK8
MAPT
MCL1
MEFV
MET
MFSD8
MID2
MLST8
MT3
MTCL1
MTDH
MTM1
MTMR3
MTMR4
MTMR7
MTMR9
MTOR
NEDD4
NLRP6
NOD1
NOD2
NPC1
NPRL2
NRBP2
NUPR1
OPTN
ORMDL3
OSBPL7
PAFAH1B2
PARK7
PHB2
PHF23
PIK3C2A
PIK3CA
PIK3CB
PIK3R2
PIM2
PINK1
PIP4K2A
PIP4K2B
PIP4K2C
PLEKHF1
PLK2
PLK3
POLDIP2
PRKAB1

PRKAB2
PRKACA
PRKAG1
PRKAG2
PRKAG3
PRKD1
PRKN
PSAP
PTPN22
PYCARD
QSOX1
RAB39B
RAB3GAP1
RAB3GAP2
RAB8A
RALB
RASIP1
RB1CC1
RHEB
RIPK2
RMC1
RNF152
RNF41
RNF5
ROCK1
RPTOR
RRAGA
RRAGB
RRAGC
RRAGD
RUBCN
RUFY4
SCFD1
SCOC
SEC22B
SESN1
SESN2
SESN3
SH3BP4
SH3GLB1
SIRT1
SIRT2
SLC38A9
SMCR8
SMG1
SNCA
SNRNP70

SNX32
SNX5
SNX6
SOGA1
SOGA3
SPTLC1
SPTLC2
SREBF1
SREBF2
STAT3
STING1
STK11
SVIP
TAB2
TAB3
TBC1D14
TBC1D25
TBK1
TFEB
TICAM1
TIGAR
TLK2
TMEM150A
TMEM150B
TMEM150C
TMEM39A
TMEM39B
TMEM59
TP53
TPCN1
TPCN2
TREM2
TRIB3
TRIM13
TRIM14
TRIM21
TRIM5
TRIM27
TRIM38
TRIM6
TRIM65
TRIM68
TRIM8
TRIML1
TRIML2
TSC1
TSC2

TSPO
UBQLN1
UBQLN2
UBQLN4
UCHL1
UFL1
USP10
USP13
USP30
USP33
USP36
UVRAG
VDAC1
VPS13C
VPS13D
VPS26A
VPS26B
VPS35
WAC
WASHC1
WDR24
WDR41
WDR6
ZC3H12A
ZKSCAN3
ZMPSTE24
ATP13A2
BNIP3
BNIP3L
CAMKK2
CSNK2A2
CTTN
DNM1L
FBXO7
FBXW7
FZD5
GSK3A
HAX1
HDAC6
HIF1A
HTRA2
HTT
PARK7
PINK1
PRKN
RNF41
SREBF1

SREBF2
TIGAR
TP53
TSC2
TSPO
USP30
USP36
VDAC1
VPS13C
VPS13D
ATP5IF1
CDC37
GBA
HDAC6
HK2
HTRA2
HUWE1
MFN2
MUL1
OPTN
PINK1
PRKN
TOMM7
VPS13C
ATP13A2
CTSA
EEF1A1
EEF1A2
GFAP
PLK3
SNCA
SNRNP70
ADRB2
AKT1
ATG101
ATG13
ATP13A2
ATP6V0A1
ATP6V0A2
ATP6V0B
ATP6V0C
ATP6V0D1
ATP6V0D2
ATP6V0E1
ATP6V0E2
ATP6V1A
ATP6V1B1

ATP6V1B2
ATP6V1C1
ATP6V1C2
ATP6V1D
ATP6V1E1
ATP6V1E2
ATP6V1G1
ATP6V1G2
ATP6V1H
BAG3
BECN1
BNIP3
BNIP3L
C9orf72
CALCOCO2
CAPN1
CAPNS1
CASP3
CDK5
CDK5R1
CHMP4B
CLEC16A
CLN3
DCN
KIAA1324
EPM2A
ERN1
EXOC1
EXOC4
EXOC7
EXOC8
FEZ1
FEZ2
FYCO1
GAPDH
GBA
GNAI3
GPSM1
HDAC6
HIF1A
HMOX1
HSPB8
HTT
IKBKG
IL4
IRGM
KDR

LAMTOR1
LAMTOR2
LAMTOR3
LAMTOR4
LAMTOR5
LARP1
LRRK2
LRSAM1
LZTS1
MAP3K7
MAPK3
MAPK8
MLST8
MTM1
MTMR7
MTOR
NEDD4
NOD1
NOD2
NPC1
NRBP2
NUPR1
OPTN
PAFAH1B2
PHF23
PIK3CA
PINK1
PIP4K2A
PIP4K2B
PIP4K2C
POLDIP2
PRKAA1
PRKAA2
PRKAB1
PRKAB2
PRKACA
PRKAG1
PRKAG2
PRKAG3
QSOX1
RAB3GAP1
RAB3GAP2
RALB
RB1CC1
RHEB
RIPK2
RNF41

RPTOR
RRAGA
RRAGB
RRAGC
RRAGD
RUBCN
RUFY4
SCFD1
SCOC
SEC22B
SESN1
SESN2
SESN3
SH3GLB1
SIRT1
SLC38A9
SMCR8
SMG1
SNX32
SNX5
SNX6
SPTLC1
SPTLC2
STING1
TBC1D25
TBK1
TIGAR
TMEM39A
TMEM39B
TP53
TRIM13
TSC1
TSC2
UBQLN1
UBQLN2
UBQLN4
UCHL1
ULK1
USP30
USP36
UVRAG
VDAC1
VPS13C
VPS13D
VPS26A
VPS26B
VPS35

WAC
ADRB2
AMBRA1
ATG13
ATG14
ATG2A
ATG2B
AUP1
BAG3
BECN1
CALCOCO2
CDC37
DDRGK1
HSPB8
HTRA2
HTT
HUWE1
KLHL3
LGALS8
LRSAM1
MAPK3
NOD1
NOD2
OPTN
PHB2
PJVK
RAB7A
RB1CC1
RETREG1
RETREG3
RIPK2
RNF41
SESN2
SPTLC1
SPTLC2
STBD1
STING1
TBK1
TEX264
TIGAR
TP53
TSC2
UBA5
UBQLN1
UFC1
UFL1
UFM1

VPS13C
VPS13D
WDFY3
WDR81

Apoptosis	Extracellular Matrix	Oxidative stress
WIPI2	COL6A2	ABCD1
ADD1	COL6A1	ABL1
AIFM3	COL6A3	ACOX2
ANK1	COL1A1	ADA
ANXA1	COL2A1	ADAM9
APP	COL1A2	ADIPOQ
ATF3	COL3A1	ABCC2
AVPR1A	COL7A1	ADNP2
BAX	COL5A1	ADPRS
BCAP31	COL9A1	AGAP3
BCL10	COL9A2	AIF1
BCL2L1	COL9A3	AIFM2
BCL2L10	COL4A3	AKR1C3
BCL2L11	COL12A1	AKT1
BCL2L2	COL5A2	ALAD
BGN	COL4A5	ALDH3B1
BID	COL4A1	ALOX5
BIK	COL4A4	ANGPTL7
BIRC3	COL10A1	ANKRD2
BMF	COL17A1	ANKZF1
BMP2	COL4A2	ANXA1
BNIP3L	COL18A1	APEX1
BRCA1	COL11A2	APOA4
BTG2	COL11A1	APOD
BTG3	COL4A6	APOE
CASP1	COL14A1	APP
CASP2	COL8A2	APTX
CASP3	COL16A1	AQP1
CASP4	COL19A1	ARG1
CASP6	COL5A3	ARL6IP5
CASP7	COL15A1	ARNT
CASP8	COL8A1	ARNTL
CASP9	COL13A1	ATF4
CAV1	COL6A5	ATG7
CCNA1	COL27A1	ATOX1
CCND1	COL25A1	ATP13A2
CCND2	COL21A1	ATP2A2
CD14	COL22A1	ATP7A
CD2	COL20A1	ATRN
CD38	COL6A6	AXL
CD44	COL24A1	BAD
CD69	COL23A1	BAG5
CDC25B	COL28A1	BAK1
CDK2	COL26A1	BCL2
CDKN1A	LAMA3	BECN1
CDKN1B	LAMC2	BMP7

CFLAR	LAMB3	BNIP3
CLU	COL7A1	BRF2
CREBBP	LAMA1	BTK
CTH	LAMB1	C19orf12
CTNNB1	LAMC1	CA3
CYLD	COL18A1	CAMKK2
DAP	LAMA2	CAPN2
DAP3	LAMA5	CASP3
DCN	LAMB2	CAT
DDIT3	LAMA4	CBX8
DFFA	ITGA6	CCL19
DIABLO	ITGB1	CCNA2
DNAJA1	LAMC3	CCR7
DNAJC3	HSPG2	CCS
DNM1L	NID1	CD36
DPYD	NID2	CD38
EBP	COL4A2	CDK2
EGR3	COL4A1	CFLAR
EMP1	COL4A4	CHCHD2
ENO2	COL4A5	CHD6
ERBB2	COL4A3	CHRNA4
ERBB3	COL4A6	CHUK
EREG	ITGA2	CLN8
ETF1	ITGAV	COA8
F2	ITGA7	COL1A1
F2R	ITGA1	CPEB2
FAS	ITGB4	CRK
FASLG	ITGA3	CRYAB
FDXR	COL9A2	CRYGD
FEZ1	COL9A1	CYBA
GADD45A	COL9A3	CYBB
GADD45B	ITGA11	CYCS
GCH1	ITGA4	CYGB
GNA15	COL1A2	CYP1B1
GPX3	COL1A1	CYP2E1
GPX4	ITGA10	DAPK1
GSN	COL2A1	DGKK
GSR	KDR	DHCR24
GSTM2	ITGB3	DHFR
H1-0	ITGAM	DHFRP1
HGF	ITGB2	DHRS2
HMGB2	JAM3	DIABLO
HMOX1	ITGB5	DNM2
HSPB1	SPP1	DPEP1
IER3	F11R	DUOX1
IFITM3	ITGAL	DUOX2
IFNB1	CD44	DUSP1

IFNGR1	IBSP	ECT2
IGF2R	TNC	EDN1
IGFBP6	VWF	EEF2
IL18	ITGA9	EGFR
IL1A	ITGA5	EGLN1
IL1B	ITGAX	EIF2S1
IL6	JAM2	ENDOG
IRF1	THBS1	EPAS1
ISG20	VCAM1	EPX
JUN	FBN1	ERCC1
KRT18	CDH1	ERCC2
LEF1	ITGB7	ERCC3
LGALS3	ITGAE	ERCC6
LMNA	AGRN	ERCC6L2
LUM	ITGA8	ERCC8
MADD	COL13A1	ERMP1
MCL1	COL10A1	ERN1
MGMT	CD47	ERO1A
MMP2	COMP	ETFDH
NEDD9	LUM	ETS1
NEFH	COL8A1	ETV5
PAK1	COL8A2	EZH2
PDCD4	COL3A1	FABP1
PDGFRB	COL16A1	FANCC
PLAT	COL6A5	FANCD2
PLCB2	COL6A6	FBLN5
PLPPR4	COL6A3	FBXO7
PMAIP1	COL6A1	FBXW7
PPP2R5B	COL6A2	FER
PPP3R1	COL5A3	FGF8
PPT1	COL5A2	FKBP1B
PRF1	COL5A1	FOS
PSEN1	ITGA2B	FOSL1
PSEN2	FN1	FOXO1
PTK2	BSG	FOXO3
RARA	FGA	FUT8
RELA	FGB	FXN
RETSAT	FGG	FYN
RHOB	VTN	G6PD
RHOT2	ITGB8	GATA4
RNASEL	ICAM1	GCH1
ROCK1	ICAM3	GCLC
SAT1	ICAM4	GCLM
SATB1	ICAM5	GGT7
SC5D	ICAM2	GJB2
SLC20A1	ITGAD	GLRX2
SMAD7	PECAM1	GNAO1

SOD1	COL23A1	GPR37
SOD2	ITGB6	GPR37L1
SPTAN1	MADCAM1	GPX1
SQSTM1	SCUBE3	GPX2
TAP1	SCUBE1	GPX3
TGFB2	MMP2	GPX4
TGFBR3	MMP13	GPX5
TIMP1	MMP9	GPX6
TIMP2	MMP12	GPX7
TIMP3	FBN3	GPX8
TNF	FBN2	GSKIP
TNFRSF12A	CTSS	GSR
TNFSF10	MMP7	GSS
TOP2A	MMP3	GSTP1
TSPO	CTSK	GUCY1B1
TXNIP	CTSV	H19
VDAC2	ADAMTS18	HAO1
WEE1	ADAMTS1	HBA1
XIAP	ADAMTS16	HBA2
CCR5	ADAMTS9	HBB
CD247	ADAMTS8	HDAC2
CD28	ADAMTS4	HDAC6
CD3D	ADAMTS5	HGF
CD3E	ACAN	HIF1A
CD3G	MMP19	HMOX1
CD4	PLG	HMOX2
FAS	CTSG	HNRNPD
FASLG	COL11A1	HNRNPM
AIFM1	COL11A2	HP
AKT1	CTSL	HSF1
AKT2	CTSB	HSPA1A
AKT3	MMP20	HSPA1B
APAF1	MMP10	HSPB1
ATM	MMP1	HTRA2
BAD	TMPRSS6	HYAL1
BCL2	MMP14	HYAL2
BIRC2	MMP8	IDH1
CAPN1	COL19A1	IL10
CAPN2	PRSS2	IL18RAP
CHP1	ADAM17	IL6
CHP2	ADAM10	IMPACT
CHUK	ADAM9	INS
CYCS	COL17A1	IPCEF1
DFFB	ELANE	JAK2
ENDOD1	CTSD	JUN
ENDOG	MMP15	KCNA5
EXOG	FURIN	KCNC2

FADD	COL12A1	KDM6B
IKBKB	PHYKPL	KEAP1
IKBKG	COL25A1	KLF2
IL1R1	COL26A1	KLF4
IL1RAP	COL15A1	KRT1
IL3	MMP11	LANCL1
IL3RA	COL14A1	LDHA
IRAK1	A2M	LIAS
IRAK2	ELN	LONP1
IRAK3	ADAM8	LPO
IRAK4	TLL2	LRRK2
MAP3K14	TLL1	MACROH2A1
MYD88	BMP1	MAP1LC3A
NFKB1	MMP25	MAP3K5
NFKBIA	MMP24	MAPK1
NGF	MMP17	MAPK13
NTRK1	MMP16	MAPK3
PIK3CA	PRSS1	MAPK7
PIK3CB	CTRB1	MAPK8
PIK3CD	KLKB1	MAPK9
PIK3CG	KLK2	MAPKAP1
PIK3R1	TIMP2	MAPT
PIK3R2	CMA1	MB
PIK3R3	TPSAB1	MBL2
PIK3R5	SPOCK3	MCL1
PPP3CA	TIMP1	MCTP1
PPP3CB	CTRB2	MEAK7
PPP3CC	OPTC	MELK
PPP3R2	NCSTN	MET
PRKACA	PSEN1	MGAT3
PRKACB	HTRA1	MGST1
PRKAR1A	CAPNS2	MICB
PRKAR1B	CAPNS1	MIR103A1
PRKAR2A	CAPN9	MIR107
PRKAR2B	CAPN14	MIR132
PRKX	CAPN5	MIR133A1
RIPK1	CAPN3	MIR17
TNFRSF10B	CAPN10	MIR195
TNFRSF1A	CAPN11	MIR19A
TP53	CAPN13	MIR21
TRADD	CAPN15	MIR29B1
TRAF2	CAPN6	MIR34A
ACIN1	CAPN8	MIR675
APC	CAPN7	MIR92A1
APIP	CAPN12	MIRLET7B
APPL1	CAPN2	MMP14
ARHGAP10	CAPN1	MMP2

AVEN	CAST	MMP3
BAK1	BCAN	MMP9
BBC3	CASP3	MPO
BMX	ADAM15	MPV17
C1QBP	DCN	MSRA
CDH1	KLK7	MSRB2
CDKN2A	TGFB1	MSRB3
CLSPN	LTBP4	MT-CO1
DAPK1	LTBP3	MT-ND1
DAPK2	LTBP2	MT-ND3
DAPK3	LTBP1	MT-ND5
DBNL	MFAP3	MT-ND6
DCC	MFAP1	MT3
DSG2	MFAP4	MTF1
DSG3	EMILIN3	MTR
DSP	EMILIN2	MYB
DYNLL1	EMILIN1	MYEF2
DYNLL2	MFAP2	NAPRT
E2F1	MFAP5	NCF1
FNTA	EFEMP1	NCF2
GAS2	FBLN2	NCF4
GSDMD	FBLN1	NCOA7
GSDME	FBLN5	NDUFA12
GZMB	EFEMP2	NDUFA6
H1-1	TGFB2	NDUFB4
H1-2	TGFB3	NDUFS2
H1-3	GDF5	NDUFS8
H1-4	BMP2	NEIL1
H1-5	BMP4	NET1
HMGB1	BMP7	NFE2L1
KPNA1	BMP10	NFE2L2
KPNB1	LOXL3	NME2
LMNB1	LOXL2	NME5
LY96	LOXL4	NME8
MAGED1	LOXL1	NOL3
MAPK1	LOX	NONO
MAPK3	COL24A1	NOS3
MAPK8	COL27A1	NOX1
MAPT	PLEC	NOX4
NMT1	DST	NOX5
OCLN	CD151	NQO1
OMA1	PCOLCE	NR4A2
OPA1	PXDN	NR4A3
PAK2	COL28A1	NUDT1
PKP1	COL22A1	NUDT15
PLEC	COL21A1	NUDT2
PPP1R13B	COL20A1	OGG1

PRKCD	PLOD3	OSER1
PRKCQ	PLOD2	OXR1
PSMA1	PLOD1	OXSR1
PSMA2	P4HA2	P4HB
PSMA3	P4HA1	PAGE4
PSMA4	P4HA3	PARK7
PSMA5	P4HB	PARP1
PSMA6	ADAMTS3	PAWR
PSMA7	ADAMTS2	PAX2
PSMA8	ADAMTS14	PCGF2
PSMB1	P3H1	PCNA
PSMB10	PPIB	PDCD10
PSMB11	CRTAP	PDE8A
PSMB2	SERPINH1	PDGFD
PSMB3	P3H3	PDGFRA
PSMB4	P3H2	PDGFRB
PSMB5	COLGALT1	PDK1
PSMB6	COLGALT2	PDK2
PSMB7	PCOLCE2	PDLIM1
PSMB8	CEACAM1	PENK
PSMB9	CEACAM6	PINK1
PSMC1	CEACAM8	PJVK
PSMC2	DMD	PKD2
PSMC3	DAG1	PLA2R1
PSMC4	NRXN1	PLEKHA1
PSMC5	PDGFB	PLK3
PSMC6	FGF2	PML
PSMD1	PDGFA	PNKP
PSMD10	TTR	PNPT1
PSMD11	NTN4	PPARGC1A
PSMD12	DDR2	PPARGC1B
PSMD13	SDC1	PPIA
PSMD14	SDC4	PPIF
PSMD2	PRKCA	PPP1R15B
PSMD3	ACTN1	PPP2CB
PSMD4	TRAPPC4	PPP5C
PSMD5	SDC2	PRDX1
PSMD6	CASK	PRDX2
PSMD7	SDC3	PRDX3
PSMD8	DDR1	PRDX4
PSMD9	ADAM12	PRDX5
PSME1	ADAM19	PRDX6
PSME2	SH3PXD2A	PRKAA1
PSME3	DMP1	PRKAA2
PSME4	DSPP	PRKCD
PSMF1	SERPINE1	PRKD1
RPS27A	ASPN	PRKN

SEM1
SEPTIN4
SFN
STAT3
STK24
STK26
TFDP1
TFDP2
TICAM1
TICAM2
TJP1
TJP2
TLR4
UACA
UBA52
UBB
UBC
UNC5A
UNC5B
VIM
YWHAB
YWHAE
YWHAG
YWHAH
YWHAQ
YWHAZ
CASP3
DFFA
DFFB
H1-0
H1-1
H1-2
H1-3
H1-4
H1-5
HMGB1
HMGB2
KPNA1
KPNB1
AKT1
AKT2
AKT3
APAF1
APIP
AVEN
BAD
BAK1

FMOD
BGN
PTPRS
NCAM1
TNXB
TNN
TNR
VCAN
NCAN
LRP4
MUSK
APP
SPARC
MATN1
MATN4
MATN3
HAPLN1
HIF1A
ATF3

PRKRA
PRNP
PRODH
PRR5L
PSEN1
PSIP1
PSMB5
PTGS1
PTGS2
PTK2B
PTPRK
PTPRN
PXDN
PXDNL
PXN
PYCR1
PYCR2
PYROXD1
RACK1
RAD52
RBM11
RBPMS
RELA
REST
RGS14
RHOB
RIPK1
RIPK3
RNF112
ROMO1
RPS3
S100A7
SCARA3
SCGB1A1
SDC1
SELENOK
SELENON
SELENOP
SELENOS
SESN1
SESN2
SESN3
SETX
SFPQ
SGK2
SIGMAR1
SIN3A

BAX
BBC3
BCL2
BCL2L1
BCL2L11
BID
BMF
C1QBP
CASP3
CASP7
CASP8
CASP9
CDKN2A
CYCS
DIABLO
DYNLL1
DYNLL2
E2F1
GSDMD
GSDME
GZMB
MAPK1
MAPK3
MAPK8
NMT1
PMAIP1
PPP1R13B
PPP3CC
PPP3R1
SEPTIN4
SFN
STAT3
TFDP1
TFDP2
TP53
UACA
XIAP
YWHAB
YWHAE
YWHAG
YWHAH
YWHAQ
YWHAZ
CTSG
GSK3A
MAPK1
MAPK3
SIRPA
SIRT1
SIRT2
SLC1A1
SLC23A2
SLC25A24
SLC7A11
SLC8A1
SMPD3
SNCA
SOD1
SOD2
SOD3
SP1
SPHK1
SRC
SRXN1
STAR
STAU1
STK24
STK25
STK26
STOX1
STX2
STX4
SUMO4
TAT
TBC1D24
THG1L
TLDC2
TLR4
TLR6
TMEM161A
TNFAIP3
TOR1A
TP53
TP53INP1
TPM1
TPO
TRA2B
TRAF2
TRAP1
TREX1
TRPA1
TRPC6
TRPM2
TSC1

RNF213
SFPQ
TRIM27
BCL2L14
BCL6
BIRC5
CHM
NDRG1
PERP
PPP1R13B
RABGGTA
RABGGTB
TP53
BIRC5
BOK
CRADD
HELLS
HRK
IGF1
IGF1R
IGF2
IRF2
IRF3
IRF4
IRF5
IRF6
IRF7
LTA
MAP2K4
MAP3K1
DUSP16
MDM2
MYC
NFKBIB
NFKBIE
SCAF11
TNFRSF1B
TNFRSF21
TNFRSF25
TRAF1
TRAF3
AIFM1
AIFM2
APAF1
BAD
BAG3
BAK1
TXN
TXN2
TXNIP
TXNRD1
TXNRD2
UBE3A
UBQLN1
UCN
UCP1
UCP2
UCP3
VKORC1L1
VNN1
VRK2
WNT1
WNT16
WRN
XRCC1
ZC3H12A
ZNF277
ZNF580
ZNF622

BAX
BBC3
BCL2
BCL2L1
BCL2L10
BCL2L11
BCL2L2
BID
BIK
BIRC2
BIRC3
BIRC5
BIRC6
BIRC7
BLK
BMF
BNIP3
BOK
CAPNS1
CASP1
CASP2
CASP3
CASP4
CASP6
CASP7
CASP8
CASP9
CDKN2A
CFLAR
CRADD
CYCS
DAXX
DFFA
DFFB
DIABLO
ENDOG
FADD
FAS
FASLG
FOS
HRK
HSPA1A
HTRA2
IKBKB
IL1R1
IL1R2
IRAK1

JUN
MADD
MAP3K14
MAP3K5
MAPK3
MAPK8
MCL1
MYD88
NFKB1
NFKBIA
PIDD1
PMAIP1
PRKD1
PTPN13
PTRH2
RIPK1
SEPTIN4
TNFRSF10B
TNFRSF11B
TNFRSF1A
TNFRSF1B
TNFRSF25
TNFSF10
TOLLIP
TP53
TRADD
TRAF3
TRAF6
XIAP
AIFM1
APAF1
BID
CASP2
CASP3
CASP6
CASP7
CASP8
CASP9
CYCS
FADD
FAS
FASLG
HSPA1A
MAP3K1
DUSP16
NFKB1
RIPK1

TNFRSF1A
ABL1
AKT1
ANXA5
APOE
APP
AXIN1
BCL3
BEX3
BIRC5
CARD14
CASP7
CDKN1A
CDKN1B
CTNNA1
CUL1
CUL5
ERBB3
ERN1
ETS1
F2R
GCLC
HDAC1
HELLS
HSPA5
HSPB1
HSPD1
IER3
IL7R
JUND
MAPK1
NET1
NFKB1
NQO1
NRG1
PAK2
PKN1
PTK2
PTK2B
RIPK2
RNF7
RPS6KB1
SERBP1
SMAD7
SOCS3
SQSTM1
THBS1

TNF
TNFRSF10B
TNFRSF21
TRAF1
VAV3
VIM
YBX3
AKT1
APAF1
BAD
BAX
BBC3
BCL2
BCL2L1
BCL2L11
BID
CASP3
CASP7
CASP8
CASP9
FADD
FASLG
MAPK11
MAPK12
MAPK13
MAPK14
MCL1
TNF
AIFM1
APAF1
BAK1
BAX
BCL2
BID
CASP3
CASP6
CASP7
CASP8
CASP9
CFLAR
CYCS
DIABLO
ENDOG
FADD
FAS
FASLG
HTRA2

PRF1
AKT1
BAK1
BAX
BCL2
BCL2L1
BCL2L10
BCL2L12
BCL2L13
BCL2L14
BCL2L15
BCL2L2
BID
BOK
GSK3A
GSK3B
ITGA6
ITGB4
MCL1
MYC
PIK3CG
PTHLH
AKT1
AKT2
BIRC2
BIRC3
CASP3
CASP7
CASP8
CCL2
CCL5
CHUK
CTNNB1
FADD
GSK3B
HDAC1
IKBKB
IL6
JUN
MAP3K14
MAP3K7
MAPK1
MAPK14
MAPK3
MAPK8
MAPK9
MMP9

NFKB1
NFKB2
NFKBIA
NFKBIB
RAC1
RAF1
RELA
RELB
RIPK1
TNF
TNFRSF12A
TNFSF12
TRAF1
TRAF2
TRAF3
TRAF5
TRIM63
ACIN1
ACVR1C
APAF1
BAX
BLCAP
BOK
CAPN10
CASP3
CDK5RAP3
CECR2
CIDEA
DEDD2
DFFA
DFFB
DICER1
DNASE1L3
DNASE2B
ENDOG
ERN2
EXOG
FOXL2
GPER1
H1-0
HMGB1
HMGB2
HSF1
IL6
KPNA1
KPNB1
NMNAT1

SHARPIN
TOP2A
ACIN1
ACVR1C
AIFM3
AKT1
APAF1
BAX
BBC3
BCL2L1
BLCAP
BNIP1
BOK
CAPN10
CASP1
CASP14
CASP2
CASP3
CASP4
CASP6
CASP7
CASP8
CASP9
CDK5RAP3
CECR2
CFLAR
CIDEA
CIDEB
CIDEC
COLEC11
DEDD2
DFFA
DFFB
DICER1
DLC1
DNASE1L3
DNASE2B
DNM1L
ENDOG
ERN2
EXOG
FAP
FASLG
FOXL2
FZD3
GCG
GPER1

H1-0
HMGB1
HMGB2
HSF1
HTRA2
IL6
KPNA1
KPNB1
MADD
NMNAT1
PAK2
PLSCR1
PTGIS
RFFL
RNF34
SHARPIN
SIRT2
STK24
TAOK1
TOP2A
TP53
TRPC5
XKR4
XKR5
XKR6
XKR7
XKR8
XKR9
ZC3H12A
BCL2L1
CIDEA
DFFA
FZD3
GCG
PAK2
RFFL
RNF34
BOK
DLC1
FAP
PTGIS
SIRT2
TP53
ZC3H12A
PAK2
RFFL
RNF34

RPS3
BCL2L1
BOK
CIDEA
DFFA
DLC1
FAP
FZD3
GCG
PAK2
PTGIS
RFFL
RNF34
SIRT2
TP53
ZC3H12A
CASP14
CASP2
CASP3
CASP6
CASP7
CASP8
CASP9
CFLAR
