# Supplementary material for: Targeting EGR1-ATF3 signaling mitigates paravertebral muscle degeneration by regulating cell death and inflammaging
Source: Biol Res. 2025 Jul 28;58:52. doi: 10.1186/s40659-025-00634-1 (PMC12302741; doi:10.1186/s40659-025-00634-1)
Supplement: Supplementary file 2 — Supplementary Material 2: Supplementary Table S2. The forward primers and reverse primers of each gene used in this study. [file 40659_2025_634_MOESM2_ESM.pdf]

Table S2 The forward primers and reverse primers of each gene used in this study

**Primers' sequence**	
CDKN1A/P21-forward	TGTCCGTCAGAACCCATGC
CDKN1A/P21-reverse	AAAGTCGAAGTTCCATCGCTC
IL-6-forward	ATGCAATAACCACCCCTGAC
IL-6-reverse	AAAGCTGCGCAGAATGAGAT
Aggrecan-forward	TGTAACCCAGGCTCCAAC
Aggrecan-reverse	GCAGCCCACTTAGGTCC
EGR1-forward	CAGCAGCAGCACCTTCAAC
EGR1-reverse	GTCTCCACCAGCACCTTCTC
ATF3-forward	GTGCCGAAACAAGAAGAAGG
ATF3-reverse	TCTGAGCCTTCAGTTCAGCA
GPX4-forward	AGAGATCAAAGAGTTCGCCGC
GPX4-reverse	TCTTCATCCACTTCCACAGCG
GAPDH-forward	AGAAGGCTGGGGCTCATTTG
GAPDH-reverse	GCAGGAGGCATTGCTGATGAT
