# Supplementary material for: Targeting EGR1-ATF3 signaling mitigates paravertebral muscle degeneration by regulating cell death and inflammaging
Source: Biol Res. 2025 Jul 28;58:52. doi: 10.1186/s40659-025-00634-1 (PMC12302741; doi:10.1186/s40659-025-00634-1)
Supplement: Supplementary file 3 — Supplementary Material 3: Supplementary Table S3. The differential expression genes and miRNAs identified based on RNA sequencing. [file 40659_2025_634_MOESM3_ESM.pdf]

GeneSymbol	log2FC	FDR	SPMD-2	SPMD-3	Normal-1	Normal-2
USP9Y	-15.59820134	0	0	0	671	0
RPS4Y1	-14.85173906	0	0	0	384	0
ZFY	-13.91413521	0	0	0	205	0
CCDC63	-12.41176972	1.04139E-13	0	0	142	2
EGFL6	-12.07276263	7.85322E-09	0	0	1	3
C1orf158	-11.92207444	3.73589E-06	0	0	13	8
DDX3Y	-10.87232618	0.000149679	1	0	732	0
KDM5D	-9.907559117	3.10294E-05	2	0	1287	1
EIF1AY	-9.795438823	2.18127E-05	2	0	1062	0
CRYM	-9.077880773	0.00033164	0	1	292	649
UTY	-8.084185199	0.000437986	1	1	384	0
ROS1	-6.144293309	0.026211553	0	1	100	3
CFAP126	-5.2294321	0.030785921	11	2	579	0
ACTN3	-4.571850063	0.027815756	116	395	2765	1761
MYLK4	-4.212397624	0.016231271	22	46	712	143
PZP	-3.933547682	0.025565909	1	1	17	25
SIM1	-2.848482772	2.07003E-10	35	9	91	168
FBXL22	-2.831681565	0.003926898	7	3	36	51
GPR1	-2.656392669	0.004518983	7	2	23	43
KLHL38	-2.605950395	0	352	576	2719	3061
DCHS2	-2.422604594	2.94888E-06	7	5	18	43
LRRC30	-2.380153428	3.16613E-12	13	23	76	111
MUC3A	-2.230854964	0.014509734	10	25	113	88
DDIT4L	-2.230131394	1.3218E-05	112	396	921	1917
LMO1	-2.225495537	0.001965138	33	59	284	190
LMOD1	-2.202706531	0.000491433	215	563	2030	1294
MAB21L1	-2.170173125	0.004925331	13	5	45	47
IRS1	-2.096777886	0	268	192	967	1067
RET	-2.048651578	0.014566892	52	26	188	101
TRIL	-2.046684431	2.43783E-12	26	34	118	162
ZNF671	-2.045304304	0.010316061	15	41	156	110
MMP15	-1.998763387	7.08196E-06	69	55	239	336
INHBE	-1.987743352	0.015114386	15	8	50	58
EN1	-1.979770528	8.32667E-15	47	70	225	291
PRPH2	-1.946489204	0.013608056	108	71	345	209
FBXO32	-1.936479131	0.035563468	2844	2786	6276	17562
KCNA5	-1.921572537	0.022581824	18	17	30	106
PPARGC1B	-1.915239536	0.00592179	100	157	421	779
E2F8	-1.914518301	0.000545193	145	143	270	714
SLC45A3	-1.914236442	1.21371E-06	28	42	152	131
INKA1	-1.894348852	0.04465102	5	19	54	63
UCP3	-1.893531455	0.004376272	341	691	1098	2186
CDKN2C	-1.886357266	0.024966542	90	78	399	270
PAK1	-1.872280945	9.91679E-06	192	179	638	523
ITLN1	-1.853828274	0.023628754	8	6	16	23
ZNF792	-1.834109929	1.96617E-05	7	20	42	70
SKP2	-1.81144208	1.79723E-05	44	26	117	160
FAM240C	-1.799321402	2.09873E-06	17	49	120	109
TPX2	-1.770206023	7.33956E-05	34	20	56	96
ACOT1	-1.750550628	0.000302784	31	27	73	84
ZNF835	-1.725233519	0.001940739	5	15	23	50
HOXC4	-1.717777222	1.29563E-13	34	28	92	129
PPP1R3A	-1.678355959	3.25142E-06	1380	1609	3549	4645
HOXA9	-1.672993862	0.00014993	50	43	131	212
ACE	-1.658324978	0.000322838	57	52	165	238
KLHL31	-1.63458381	0	1401	1214	3473	5025
DBNDD1	-1.627581769	2.10151E-07	199	368	884	816

ASB11	-1.613086746	2.69328E-06	500	455	1308	1214
AGAP2	-1.596924305	0.002018207	84	85	187	395
CMBL	-1.596567171	3.17539E-08	624	1165	2193	2847
SCN4B	-1.591661938	1.58878E-06	229	244	503	1025
HOXC6	-1.526851304	1.48976E-10	142	171	446	548
MAP10	-1.515335783	2.34219E-06	27	18	58	83
ISLR2	-1.498364194	7.30281E-06	24	18	63	60
PARS2	-1.492393904	2.98519E-07	25	24	73	71
P2RY1	-1.490253474	0.028543764	43	54	88	130
SIRT4	-1.485955696	0.001649575	31	22	84	74
NDRG4	-1.477034721	0.002129219	48	73	115	257
LBX1	-1.450539623	0.000443216	41	26	85	121
PAX7	-1.443352664	0.038574593	104	63	209	323
PDZRN3	-1.428244946	0	365	429	902	1220
HS3ST5	-1.427224183	5.35878E-11	49	41	122	133
TMTC4	-1.424992047	0	104	67	211	226
HSPA12B	-1.422090508	0.028632246	76	128	263	404
SLC40A1	-1.409908872	0.001281082	948	923	1488	3529
ZNF174	-1.407830555	3.52741E-06	53	33	106	143
ZNF555	-1.403737245	0.017790878	50	45	133	164
ZNF253	-1.403500848	0.001845433	44	83	124	250
NEDD1	-1.399998232	4.29212E-13	294	277	751	854
ZNF607	-1.376207016	1.91167E-05	17	26	43	61
KBTBD13	-1.367927448	2.56895E-06	107	143	340	306
WNT5A	-1.363110904	0.009530606	59	82	156	272
DOLK	-1.357063679	0.021941235	47	30	114	93
CHAC1	-1.34925018	0.014198128	23	97	128	179
ACOT2	-1.347330846	0.000466413	219	338	789	737
MFSD9	-1.344623344	0.002713922	26	56	92	148
PPP1R3B	-1.32390287	4.84152E-08	1205	1396	2801	4354
ASB2	-1.318560132	2.68008E-13	2372	3240	6658	7317
CCDC69	-1.305841555	4.4181E-06	1773	1799	3723	4032
DYRK2	-1.303256458	0.006054802	734	268	906	1627
BTNL9	-1.284004331	0.002830037	268	455	811	1224
ZNF449	-1.2609892	0.002067073	70	78	178	232
HOXB2	-1.258639337	1.05125E-11	74	76	160	227
ZNF30	-1.239458037	0.044151858	26	24	67	67
LRRC38	-1.236067191	0.00130991	118	245	436	434
HEY2	-1.235471839	2.64011E-13	91	69	185	187
BMPER	-1.224119306	0.043769872	30	64	74	162
AKR1C2	-1.223960114	2.63413E-08	911	438	1214	1599
GPT2	-1.221959673	0.028977871	1608	2154	4995	4099
DENND2A	-1.218108483	0.007425082	98	63	180	243
HOXB3	-1.216935984	7.12777E-06	84	96	197	270
EHHADH	-1.215172623	0.007782672	22	39	60	68
MAF	-1.213567219	1.96377E-07	557	593	1366	1271
RBM43	-1.212060554	0.000626243	48	39	85	140
NDUFA4L2	-1.205066599	1.28878E-08	157	144	236	438
PLAAT1	-1.199651439	0.000281907	189	172	452	431
TET1	-1.198895971	0.025183935	31	23	35	87
ZNF169	-1.185371248	0.029880961	17	25	51	59
IRF2	-1.183404368	0	181	195	358	471
ATP23	-1.176768721	0.005604555	50	63	139	149
SLC6A1	-1.170509715	4.7961E-07	42	59	97	119
ASB16	-1.158501964	5.27411E-06	365	326	649	705
KBTBD6	-1.15650577	0.000337953	72	46	116	172
HJV	-1.152798815	5.32907E-15	1851	2279	3762	5150
HOXB4	-1.14978681	0.027523046	34	51	94	127

CCDC112	-1.149708157	3.30464E-05	56	37	89	134
AMIGO1	-1.146846038	0.000283438	235	213	534	459
KANK3	-1.144045875	0.04441963	76	106	152	305
TSHZ1	-1.139768778	0.012150324	229	177	289	648
AFAP1L1	-1.126800118	0.004875275	387	440	834	765
SETD1B	-1.121543318	3.86725E-06	244	223	532	531
STRADB	-1.120299612	1.11606E-06	501	665	941	1497
NRG4	-1.12012069	0.015445723	121	79	126	295
SLC20A2	-1.111214091	0.002841717	2339	1907	3580	6462
IMPA2	-1.110660833	0.017295681	889	749	1874	1449
TRUB1	-1.109228686	5.28453E-07	116	89	205	274
ARHGEF37	-1.10769755	4.49412E-05	499	323	676	847
ZXDA	-1.10734327	0.002425857	62	49	122	144
ZADH2	-1.107019188	9.8993E-09	439	627	959	1392
RASL12	-1.102599238	0	110	118	215	289
BHLHE41	-1.102468006	0	767	725	1254	1834
TIGD5	-1.100818393	0.000339955	49	31	74	76
MIA2	-1.098306809	0	417	401	747	931
MFNG	-1.094083507	0.048657719	54	37	93	129
SIX4	-1.093725331	0.016225754	402	409	633	1271
CDKN1B	-1.086739637	0.010795594	461	663	1208	1502
GPD2	-1.086661588	0.000126299	282	450	596	941
EIF4EBP1	-1.085492242	0.032661067	81	146	233	321
KLF8	-1.084497069	0.017667241	80	94	151	269
AMPD1	-1.078734313	2.1795E-07	5167	5815	10382	11220
QPRT	-1.077789864	0.042871149	54	98	176	163
ARHGAP20	-1.073754886	0.039279263	22	26	35	74
ZNF395	-1.06947502	2.33115E-11	132	125	195	320
MGME1	-1.068859016	0.001046532	88	158	209	329
DDI2	-1.068670231	4.26731E-11	715	630	1019	1783
FLT4	-1.059533149	3.71229E-09	59	73	113	171
KBTBD12	-1.05867168	0.033575941	866	764	1845	1435
CLDN5	-1.054224642	0	205	172	372	430
AGL	-1.051693721	0	4512	5239	8905	11357
FAM220A	-1.038324127	0	476	422	730	1156
GPR180	-1.036315093	1.11022E-16	110	103	166	263
SLC43A1	-1.03448521	2.93083E-08	110	124	230	281
PHTF2	-1.033169287	4.15241E-06	2221	1318	2598	3872
RGS14	-1.031743025	0.025688677	91	37	113	159
ZNF76	-1.031167092	0.035518838	394	446	920	732
AIMP2	-1.030074624	4.02008E-05	333	384	666	685
TCEA3	-1.029091899	0.022999433	1903	3227	5360	5482
PRR12	-1.027464597	0.017764641	180	336	534	588
PPP1R1A	-1.026310143	0.038740937	1927	3590	5137	5810
THAP8	-1.024178529	0.015724211	42	39	91	80
ZFP30	-1.023942136	0.000517442	61	61	123	152
TOB1	-1.02391436	1.6299E-07	840	1138	1637	2309
RPUSD2	-1.023885065	0.023096138	63	52	128	127
BLCAP	-1.021656205	1.02029E-07	1658	1951	3213	4631
THEM6	-1.019578482	0.003379147	72	92	174	156
TMEM131L	-1.017920737	0.02203102	579	573	766	1668
CAND2	-1.015809845	0.000238714	1466	1330	2927	3159
ZNF688	-1.014603515	0.010400015	85	78	182	163
CPAMD8	-1.014523608	1.75617E-08	115	96	213	228
NUDT12	-1.011975053	1.11022E-16	152	177	290	376
HDAC9	-1.010719912	3.7366E-09	265	199	332	588
GUCD1	-1.007356466	1.71006E-05	931	1000	1843	1802
ASB12	-1.001266725	0.004972854	179	245	450	457

DNAJB4	1.010274525	0.000104238	7066	5202	1737	3227
NET1	1.01531676	0.006313299	2186	2079	528	1401
DDX3X	1.015348842	0.008233759	11147	10483	2706	7038
THOC6	1.034038053	5.56093E-07	340	334	156	145
RARB	1.03482538	0.022124955	239	170	55	165
H2AZ1	1.041846629	7.17825E-05	775	775	238	450
ID4	1.041906139	0.025774983	277	260	53	178
BCR	1.046602112	0.000205552	646	412	149	300
IGSF11	1.047719259	0	603	464	221	320
DDX5	1.051777448	3.47303E-05	17928	14378	4832	7415
FRMD8	1.054905717	5.76393E-06	431	291	185	131
CPEB4	1.064623109	8.17637E-10	5132	4820	1615	3045
PRNP	1.067976539	2.26071E-08	2934	1968	815	1202
RLIM	1.069664161	1.12632E-12	1464	1281	430	722
RAB8B	1.078573038	0.027111799	592	372	97	300
NDRG1	1.088887163	0.008940241	3896	2554	757	1636
TLNRD1	1.09336183	0.02340157	347	209	80	111
SESN2	1.093621125	0.000916584	224	205	78	71
AACS	1.094507503	0.000514348	296	200	128	70
SLC22A4	1.109615177	0.006546691	216	140	39	88
FST	1.135380165	0.040909852	295	176	43	142
NOS3	1.137060528	0.000831768	363	267	59	213
INTS6	1.140829922	1.12785E-11	1063	984	310	521
RALGDS	1.142278618	0.009638712	242	215	78	61
RRAGC	1.144536808	6.44517E-05	445	298	105	153
SERPINF2	1.151100071	2.89736E-07	110	85	38	33
MAP3K6	1.152157433	8.88178E-16	947	730	375	297
RIPK1	1.157575352	0	946	753	286	381
FBXO5	1.17354753	0.012682035	111	63	20	44
PTPN13	1.181751336	0.033537979	560	321	87	265
TXLNG	1.182606674	1.10356E-13	579	504	145	322
PCOLCE	1.185859677	0.000100252	1174	685	364	366
RBMXL1	1.186518361	4.23076E-08	530	511	158	231
PEG10	1.191115046	0.011280243	425	457	104	292
IER5L	1.192300165	0.000504469	110	74	17	51
STING1	1.194111216	5.96814E-07	971	628	212	393
GPR75	1.200881601	0.034942176	75	61	11	27
FZD5	1.217280705	3.01868E-05	200	160	67	73
ELL	1.218961511	8.54958E-08	916	894	373	306
ADAM22	1.22151238	0.011100744	268	146	51	116
RAB20	1.222588884	1.07461E-08	116	82	27	43
TXLNA	1.227578094	3.04619E-08	1626	1054	579	460
SRPX	1.231152444	0	327	235	110	114
AOC2	1.237379231	0.01908421	40	41	13	13
TAP1	1.237454238	0.005559015	1187	1137	237	473
PLK2	1.237589009	0.014629025	687	429	83	336
ZNF331	1.238061513	0.004227575	867	480	197	271
SLC38A1	1.239256567	0.000976035	622	700	194	332
COL24A1	1.243706167	0.007633839	110	117	23	53
TRIM22	1.247403047	0.010604666	1585	845	349	571
FADS3	1.247804288	3.33067E-16	1123	790	416	346
SEMA4A	1.249970357	1.15494E-05	297	176	69	108
PLB1	1.259817243	4.81524E-08	108	92	45	26
RANBP3L	1.260511352	0.0010552	83	47	18	36
HSP90AA1	1.267135016	9.36917E-13	45131	34558	10926	15610
PLEKHO2	1.26871674	0.003338611	510	456	82	197
BDNF	1.29485552	0.008666155	74	42	13	36
ETS2	1.305196884	0.020471534	5131	4866	730	2171

DLEU7	1.305533974	0.020493171	74	40	12	23
NOLC1	1.313682146	2.27833E-07	3561	2270	836	1064
BEST1	1.318690845	0.001491883	206	173	32	67
ARID5A	1.318814153	0.026595105	1451	1288	247	413
ERF	1.327459014	0	563	403	117	224
ABCG2	1.33088217	0.002129421	67	65	15	20
CES4A	1.332549621	1.86519E-05	203	225	84	86
TNFRSF10A	1.333740982	0.017860485	109	73	8	60
SMCO4	1.334081256	9.84916E-07	92	55	22	34
HSPE1	1.348481044	0.005880935	300	192	28	117
DACT1	1.354649841	6.32706E-05	353	256	51	116
PLEKHG1	1.357207063	1.66178E-06	612	455	79	264
EFNA5	1.358601975	0.014721977	70	53	12	14
URB2	1.363952923	0	639	461	196	206
OLFM2	1.372520422	2.39396E-07	138	84	45	43
NOP58	1.376567481	4.16334E-14	1656	1210	311	604
EPHA2	1.378401485	0	323	239	73	133
SELP	1.386956441	0.001037232	323	295	30	176
HSPD1	1.396386055	5.86777E-05	9523	5783	1972	2483
GLDC	1.401121735	0.043018063	54	32	22	21
SAT1	1.421983984	0.00358143	4858	3363	427	1724
OSGIN1	1.422076925	0.024064579	722	405	180	89
ARHGEF28	1.426524937	1.27631E-12	221	143	45	79
EZH2	1.427765606	0.012702898	167	205	78	45
BAIAP2	1.428807807	0.002507536	291	152	53	94
SPAG1	1.442992027	0.000133925	68	40	10	24
MT1E	1.447737241	0	2334	1750	528	669
MID1	1.448512857	0	233	174	41	99
DZIP1L	1.452679663	0.00026244	133	74	27	53
SFPQ	1.454206641	0	7537	5312	1865	2212
HELZ2	1.454720831	0	802	633	190	247
TNFRSF10B	1.466946429	3.54342E-08	1084	1033	262	310
SLCO4A1	1.467080115	3.98662E-08	428	418	79	176
MAT2A	1.471789087	6.12764E-05	20921	14190	3838	4509
RGS16	1.47543555	0.010522975	816	527	47	282
F3	1.484097785	0.026124617	1017	488	237	240
LRRC32	1.485850887	7.55249E-05	1123	694	125	446
MT1X	1.49224108	5.35818E-05	3313	2913	563	842
GPR4	1.493797095	2.46605E-11	191	137	26	81
BEX2	1.499002947	0.039216825	165	106	85	20
DNAJA1	1.500251657	3.81441E-07	5833	4491	791	1763
CCN1	1.501421848	0.000215796	13891	10850	1567	4059
CNKSR3	1.505674052	0	264	183	52	101
SDC4	1.50973709	0.000207355	3349	3024	318	1287
HK2	1.517856197	0.021070624	2549	1439	498	338
EDA2R	1.522396121	0	260	190	60	101
RAB15	1.522842711	0	1135	960	237	412
RND3	1.522937969	0.027276192	2194	1344	301	351
ELN	1.526349307	0	1975	1595	602	814
OSTN	1.527701538	0.008104009	212	260	51	125
ZNF460	1.549557137	0.00098551	230	235	45	51
LDHB	1.565450095	2.06263E-05	71	53	5	35
SCN5A	1.572118345	4.36983E-06	126	71	29	40
AEN	1.572679246	0	516	345	143	130
AASS	1.572868621	2.8461E-08	1417	1515	469	431
PROSER2	1.573217993	2.63549E-06	83	65	17	13
C7	1.574743465	0.011402665	612	647	34	342
ATF3	1.581561256	0.013282084	5745	4818	144	2072

AMD1	1.598368259	4.4253E-10	2758	1765	425	789
KLHL40	1.625983167	0.045331463	18078	8859	4955	3396
ETV4	1.648781988	0.002854652	46	56	14	25
CDH19	1.659674842	0.003417833	58	49	13	36
HEPACAM	1.668534585	0.006330057	36	40	11	3
TM4SF1	1.684926569	6.45085E-05	5581	3469	374	1970
GADD45A	1.694026227	5.43457E-07	2696	2226	286	642
DCUN1D3	1.710182346	7.93263E-06	867	627	92	161
SLC16A9	1.711958696	0.013293393	188	94	13	51
CYP1B1	1.715090701	0.000725906	962	523	86	308
PER2	1.716093033	2.73297E-05	2229	2040	420	284
ENDOU	1.717780702	0.035739373	43	45	1	11
ENAH	1.720259343	1.29891E-05	12065	7122	1523	4532
SPDYA	1.726124961	0.032720964	39	18	8	4
IRS2	1.737875689	3.60865E-05	3502	2158	267	911
PAQR6	1.747472869	0.001052241	35	43	13	8
ADAM32	1.760731129	0.039902811	19	26	8	5
CKS2	1.764176315	9.85649E-05	147	76	30	36
STC2	1.764365008	0.043925155	178	256	57	82
CLDN1	1.771683052	0.000145793	46	53	8	23
NPIPB11	1.774602021	0.001151967	98	88	28	18
ADM5	1.778446564	0.026207744	29	14	4	9
FKBP11	1.780145422	2.33724E-12	186	139	35	26
BCL3	1.797986026	5.06385E-10	736	475	106	134
PAPPA	1.809754789	9.37723E-11	78	73	11	19
REC8	1.811410258	9.59811E-11	113	110	28	18
CDKN1A	1.872558409	9.5933E-06	11181	7666	600	2277
MT1M	1.878143916	0.001210911	919	532	41	171
HSPA1B	1.892592938	0.000139335	16509	11339	631	3161
IRF1	1.895903191	7.06293E-08	1798	1260	112	372
KCNK15	1.897805218	0.031019553	35	20	2	2
COL13A1	1.898258194	9.53174E-09	199	115	30	39
ARL13B	1.902813814	0.03139991	318	139	29	79
ZC3H12A	1.920499006	0.010617055	816	524	43	51
MYH3	1.931083504	1.0153E-05	8987	4884	2070	1378
PPRC1	1.95064503	1.53808E-05	2478	1452	294	282
TGIF1	1.951100695	0.001984746	1391	685	145	240
SERPINB9	1.979003676	7.62232E-06	607	344	38	139
SRSF12	1.980203384	0.020958892	29	13	5	7
EGR3	1.982102471	0.025426026	1112	519	57	209
EIF4A1	2.000580692	8.46996E-06	243	275	44	38
TRAF4	2.005091708	0.000586064	112	124	13	12
VARS2	2.007708587	0.002493198	21	24	8	0
SPAG5	2.008282572	0.042052821	95	38	14	19
ADPRHL1	2.009697798	2.03693E-12	46464	32719	7648	4122
C11orf91	2.012808468	0.00017803	44	39	2	5
RND1	2.0327747	1.35232E-08	202	206	13	60
ADAMTS9	2.036593235	2.4647E-14	2083	1356	157	583
ATP1A1	2.042456644	1.12759E-05	11474	6823	527	2244
CACNA1D	2.044650746	2.51533E-07	49	35	1	19
HLA-DQB1	2.072128976	0.036555509	112	160	60	17
PNP	2.073433327	0.000218644	1768	1309	29	185
GCH1	2.075242172	0.000317985	468	252	14	91
IL6	2.09166733	0.000418232	2538	1958	13	269
ZFAND2A	2.097506824	0.003649768	487	225	41	78
C2CD4B	2.098287077	2.55093E-05	214	246	12	89
HOGA1	2.104389817	0	153	120	38	17
HSPA1A	2.125406468	0.000949749	42293	24301	1479	5565

ARMC12	2.135889668	0.042617009	20	31	7	6
IFRD1	2.139981717	1.19592E-07	845	934	89	193
NFKBIE	2.140530232	0	153	122	33	12
MMP19	2.151387976	1.06048E-05	804	512	68	34
BATF3	2.152009651	0.000316588	89	44	9	10
COL25A1	2.15969057	1.50702E-06	73	88	16	21
TJP2	2.176137546	3.01092E-13	124	79	12	18
ELOVL7	2.185526021	0.004039029	76	91	6	45
GRHL1	2.206758355	1.34714E-11	214	127	19	50
HAPLN3	2.208521433	1.5402E-11	755	464	85	82
ERRFI1	2.217529789	0.039831175	11275	5157	538	1356
CLCNKA	2.219454355	1.07261E-07	96	54	20	7
ARRDC4	2.234232799	0	2758	2299	192	562
HBEGF	2.242966573	0.00133075	5082	2479	294	774
ADM	2.250942912	0	924	685	47	201
FOXD1	2.255374738	1.15911E-07	115	66	8	15
SELE	2.2561468	8.77076E-15	1928	1460	5	520
CDC7	2.262568656	0	90	57	8	18
ELL2	2.31011707	0.004445981	4961	2513	179	339
KRT19	2.353810677	0.015020875	13	17	3	0
SERTAD1	2.361177774	0	2234	1729	183	205
POU5F1	2.382761435	0.015510407	27	41	11	2
RGPD5	2.386926647	0.008257416	106	43	14	8
LRG1	2.417442494	1.41693E-07	242	135	6	49
NNMT	2.422071028	1.88977E-11	9985	6437	344	1203
KRT7	2.445194534	1.57081E-05	108	127	9	8
CYP27B1	2.45923615	0.000491364	31	15	4	1
KRT80	2.46282778	0	143	91	20	23
FGB	2.487170663	0.010794782	68	88	0	0
MAFF	2.523600676	3.62235E-10	4394	3800	51	306
ADAMTS4	2.563392796	1.50333E-11	3672	3290	23	339
LAG3	2.585249767	0.000418481	48	24	2	1
PCDH10	2.618328871	0.003168087	22	29	0	2
ICAM1	2.622561621	0	2354	1704	56	318
CHRNE	2.628140293	0	93	96	11	19
SLC2A1	2.738882815	9.04877E-11	400	218	35	53
DHDH	2.751773941	0.016368697	48	18	3	2
FGA	2.936809105	0.021512117	67	111	0	0
IL21R	3.074338264	0.000641895	53	44	4	3
ANKRD1	3.153444358	0	107181	74981	4640	7524
CCL2	3.180481546	0.002857954	7898	3246	63	510
ULBP2	3.332486004	3.43064E-06	48	23	1	4
PTX3	3.402968082	2.23155E-13	280	152	15	19
ICAM4	3.433711728	1.24255E-11	53	30	0	4
RAP1GAP	3.51363056	0.017020167	9	15	2	1
SLC30A3	3.614271782	0.020408725	61	19	3	4
TEX14	3.708415229	0.000541579	94	36	2	6
MYO5B	3.76369019	0.001361085	36	14	0	1
PRODH	3.942067171	0.00049448	63	24	2	4
OR52N1	4.013955287	0.010679985	8	13	0	1
SLC25A48	4.150790476	0.02529813	187	49	10	3
ARC	4.515735212	0.00828214	1361	110	12	7
PRKCG	4.686736853	0.033428131	52	3	0	2
FAM110C	4.81096968	0.000210444	42	30	0	3
ZSCAN10	4.839081127	0.000135099	23	38	0	0
TEX26	4.959106399	0.015254705	45	5	0	0
ADGRG4	5.325367954	1.5343E-06	21	10	0	0
CLEC3A	5.836107927	0.013958414	51	0	1	0

OLIG2	6.032415656	0.021175598	45	0	0	1
IL24	6.100557832	0.000880119	111	8	2	0
TAC1	6.204388398	0.000364846	451	11	0	7
CCL22	11.26749592	0.003304248	54	2	0	0

Normal-3
681
423
216
0
118
89
1576
1012
1075
1
347
1
4
13416
932
4
175
14
14
2604
33
95
45
944
154
1964
20
763
146
89
79
146
21
173
392
8676
64
285
627
103
22
2536
204
760
33
38
74
117
109
121
31
75
5740
93
102
3309
888

1625
184
3053
601
334
44
45
54
181
51
143
57
123
1017
92
209
165
2384
77
69
143
574
62
292
121
68
168
558
80
2515
6569
4936
943
634
113
140
36
406
162
104
1581
3626
114
151
82
1198
70
344
304
60
32
417
87
119
848
90
4601
62

74
417
154
380
996
394
1327
211
3421
1653
159
975
78
1071
213
1573
90
855
58
678
844
796
175
133
11987
136
43
273
243
1324
124
1513
322
9353
806
209
191
3784
94
806
767
4663
450
5800
64
87
2012
79
2993
152
1039
2057
125
169
307
436
1941
338

3781
1198
6091
169
77
418
157
286
205
10048
165
2336
1307
744
266
1948
174
135
120
109
126
152
520
151
217
52
384
422
46
211
236
408
273
186
49
395
47
80
424
88
51
549
114
23
719
274
332
300
65
527
364
105
45
24
20645
302
20
3020

30
1399
119
906
214
40
76
40
27
135
174
266
41
202
32
673
107
149
3681
3
2290
298
70
73
86
23
925
78
29
2554
309
522
194
9337
371
265
375
1747
64
22
2685
6922
75
1684
1057
66
408
1048
383
72
130
23
25
132
513
39
270
2984

915
3529
10
3
19
1780
1250
390
59
261
1106
27
2485
11
1254
12
6
26
52
13
30
5
70
247
32
43
4466
346
6898
690
16
49
66
390
1522
820
363
169
3
316
102
58
7
13
15110
22
75
487
3570
11
13
812
139
1204
113
64
32
14342

4
308
40
299
22
16
33
8
38
197
3000
16
806
1177
245
30
535
18
1540
5
688
5
16
48
2784
44
6
17
39
1650
1317
13
10
575
15
43
8
33
9
16636
1137
5
23
7
0
2
6
4
2
1
4
62
1
1
3
2
1
0

0
0
2
0

AccID	log2FC	FDR	Normal-1
hsa-miR-653-5p	-4.413468162	0.040435801	28
hsa-miR-378j	-2.94604133	0.009164145	14
hsa-miR-196a-3p	-2.562806371	0.02693524	67
hsa-miR-378f	-1.171096072	4.32057E-06	151
hsa-miR-331-3p	-1.144152486	2.56582E-10	1293
hsa-miR-4286	-1.112901271	0.025903764	113
hsa-miR-200c-3p	1.089672661	1.07457E-07	55
hsa-miR-450a-2-3p	1.346595547	0.005581647	11
hsa-miR-185-3p	1.395107016	6.67779E-06	209
hsa-miR-5698	1.410576884	3.07341E-06	60
hsa-miR-4642	1.4352701	5.13607E-06	48
hsa-miR-542-5p	2.215785329	4.93791E-05	97

Normal-2	Normal-3	SPMD-2	SPMD-3
242	29	8	3
6	19	1	3
81	39	1	28
143	185	48	125
845	1190	470	706
58	115	35	69
30	65	110	131
9	31	42	54
74	132	385	447
102	41	185	256
15	39	81	132
107	152	422	899
