# Supplementary material for: Targeting EGR1-ATF3 signaling mitigates paravertebral muscle degeneration by regulating cell death and inflammaging
Source: Biol Res. 2025 Jul 28;58:52. doi: 10.1186/s40659-025-00634-1 (PMC12302741; doi:10.1186/s40659-025-00634-1)
Supplement: Supplementary file 4 — Supplementary Material 4: Supplementary Table S4. The 81 functional differential expression genes related to PMD. [file 40659_2025_634_MOESM4_ESM.pdf]

Ferroptosis	Apoptosis	Extracellular Matrix	**Pyroptosis**	**Inflammasome**
CDKN1A	ATF3	COL13A1	IL6	LRRC30
GCH1	CDKN1A	COL25A1		LRRC38
ACOT1	CDKN1B	COL24A1		LRRC32
ETV4	DNAJA1	FGA		DDX3X
EZH2	EGR3	FGB		
SLC40A1	GADD45A	ICAM1		
SESN2	GCH1	ICAM4		
GPT2	IL6	ADAMTS9		
SETD1B	IRF1	ADAMTS4		
SLC2A1	PAK1	MMP19		
NNMT	SAT1	MMP15		
RIPK1	TAP1	ELN		
SLC38A1	RIPK1	PCOLCE		
SAT1	TNFRSF10B	SDC4		
CHAC1	TJP2			
ATF3	SFPQ			
IL6	NDRG1			
STING1	IRF2			
NDRG1	NFKBIE			
AKR1C2	HSPA1A			
	PTPN13			
	BCL3			
	HSPD1			
	NET1			
	CCL2			
	BLCAP			
	ZC3H12A			

**Autophagy**	**Senescence**	Oxidative stress	Functional DEGs
ARL13B	CDKN1A	CYP1B1	UCP3
HSP90AA1	CDKN1B	EZH2	ATF3
RRAGC	CDKN2C	GCH1	CDKN1A
IRF1	ERF	HSPA1A	CDKN1B
IRF2	ETS2	HSPA1B	DNAJA1
CDKN1A	EZH2	IL6	EGR3
CDKN1B	H2AZ1	KCNA5	GADD45A
IL24	IL6	NET1	GCH1
IL6	QPRT	NOS3	IL6
FZD5	SLC2A1	PPARGC1B	IRF1
HK2	FBXO5	PRNP	PAK1
SESN2	PLK2	PRODH	SAT1
STING1	CCN1	RGS14	TAP1
PLK2	ICAM1	RIPK1	RIPK1
ZC3H12A		SESN2	TNFRSF10B
		SFPQ	TJP2
		UCP3	SFPQ
		ZC3H12A	NDRG1
			IRF2
			NFKBIE
			HSPA1A
			PTPN13
			BCL3
			HSPD1
			NET1
			CCL2
			BLCAP
			ZC3H12A
			ACOT1
			ETV4
			EZH2
			SLC40A1
			SESN2
			GPT2
			SETD1B
			SLC2A1
			NNMT
			SLC38A1
			CHAC1
			STING1
			AKR1C2
			COL13A1
			COL25A1
			COL24A1
			FGA
			FGB

ICAM1
ICAM4
ADAMTS9
ADAMTS4
MMP19
MMP15
ELN
PCOLCE
SDC4
LRRC30
LRRC38
LRRC32
DDX3X
ARL13B
HSP90AA1
RRAGC
IL24
FZD5
HK2
PLK2
CDKN2C
ERF
ETS2
H2AZ1
QPRT
FBXO5
CCN1
CYP1B1
HSPA1B
KCNA5
NOS3
PPARGC1B
PRNP
PRODH
RGS14
