# Supplementary material for: Targeting EGR1-ATF3 signaling mitigates paravertebral muscle degeneration by regulating cell death and inflammaging
Source: Biol Res. 2025 Jul 28;58:52. doi: 10.1186/s40659-025-00634-1 (PMC12302741; doi:10.1186/s40659-025-00634-1)
Supplement: Supplementary file 5 — Supplementary Material 5 [file 40659_2025_634_MOESM5_ESM.docx]

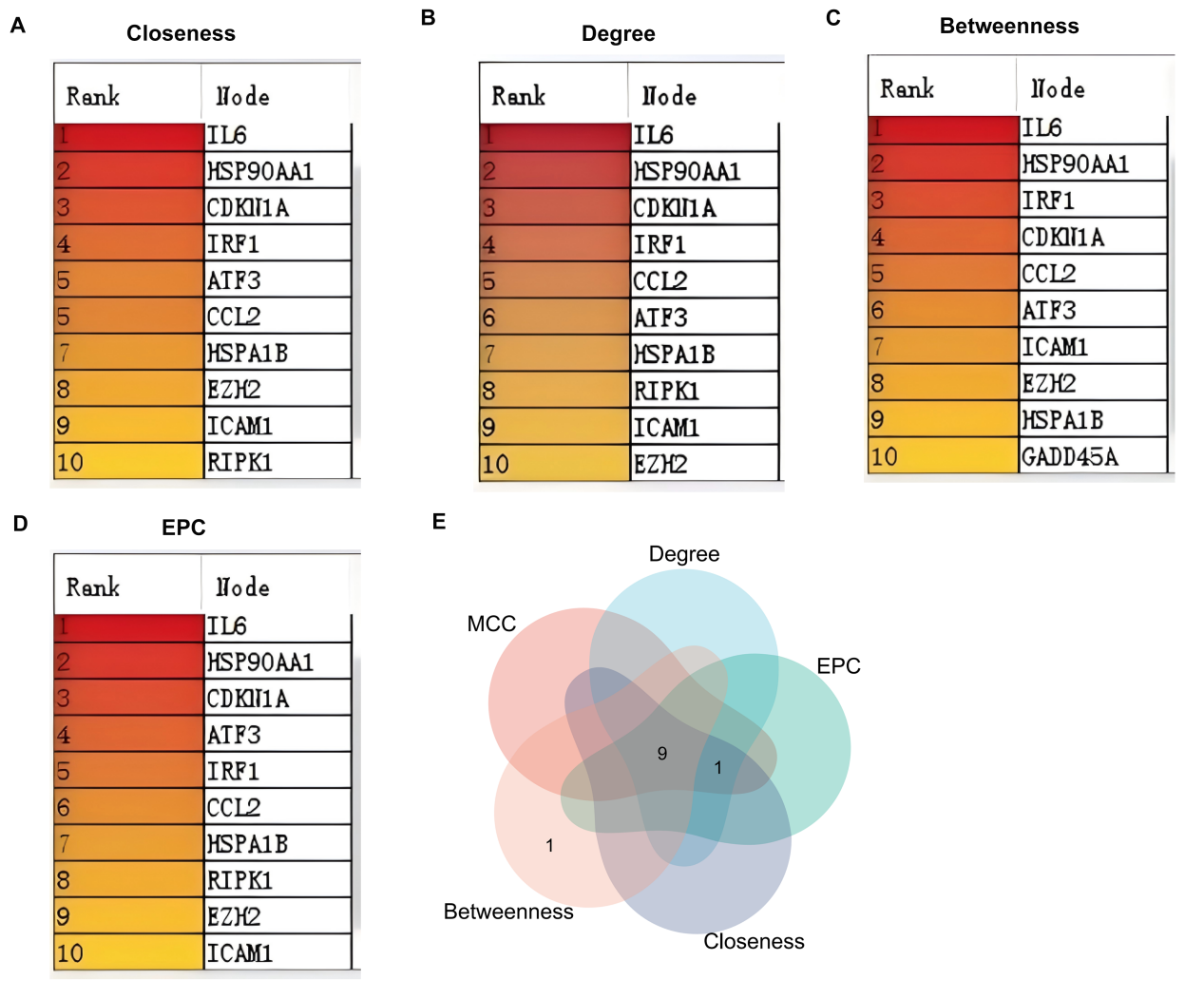


**Supplementary Figure S1. Identify the top 10 key hub genes.** A-D: CytoHubba plugin screened the top 10 hub genes using Closeness, Degree, Betweenness, and EPC algorithms. E: Venn diagrams are used to screen for overlapping hub genes.


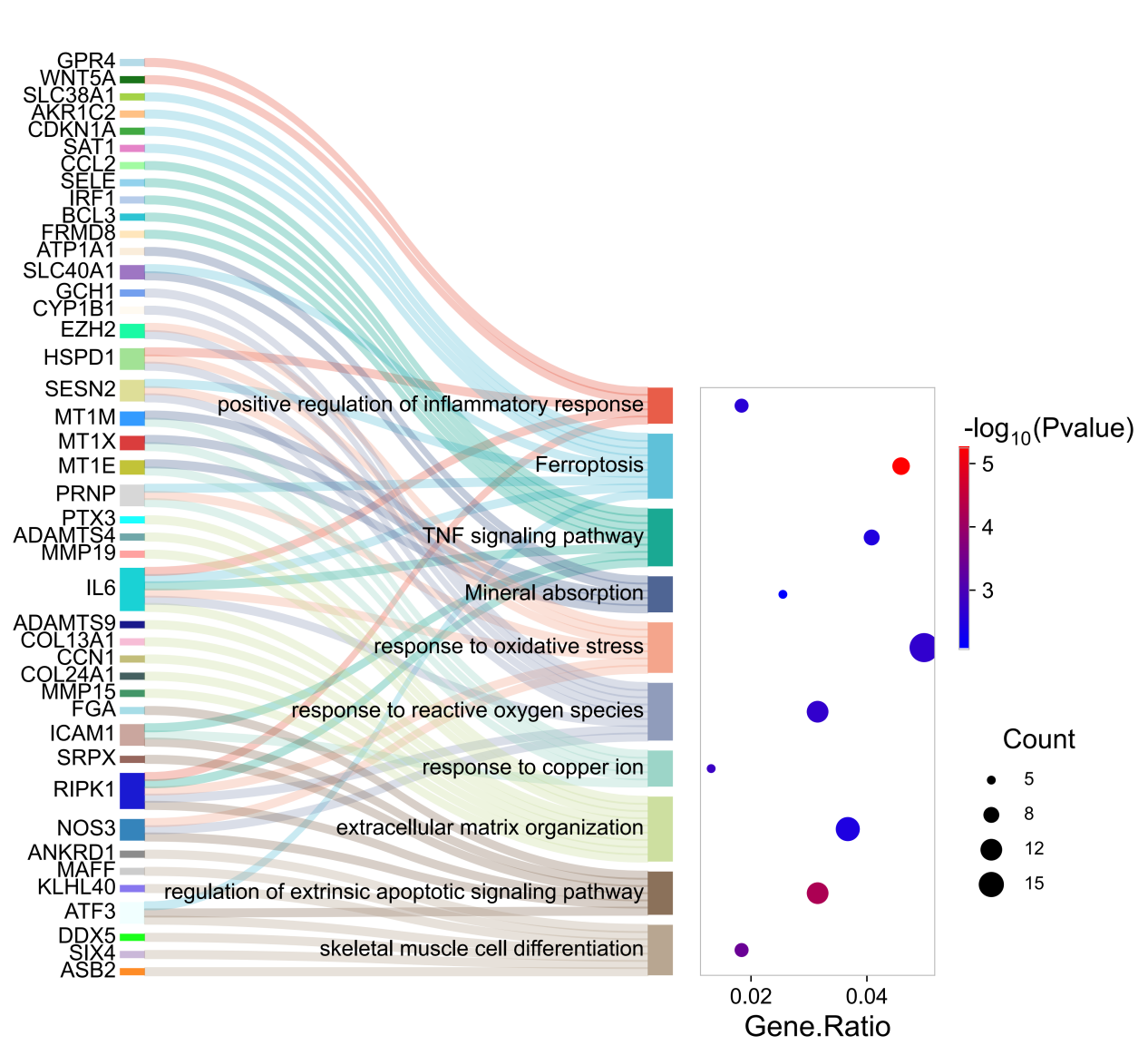


**Supplementary Figure S2. Go functional annotation and KEGG pathway enrichment analysis of all the 409 DEGs**. Sankey and bubble diagram. The left is a sankey diagram, indicating the DEGs linked to each pathway; the right is a bubble diagram, the bubble size indicates the number of DEGs corresponding to each pathway, and the bubble color indicates the p value.


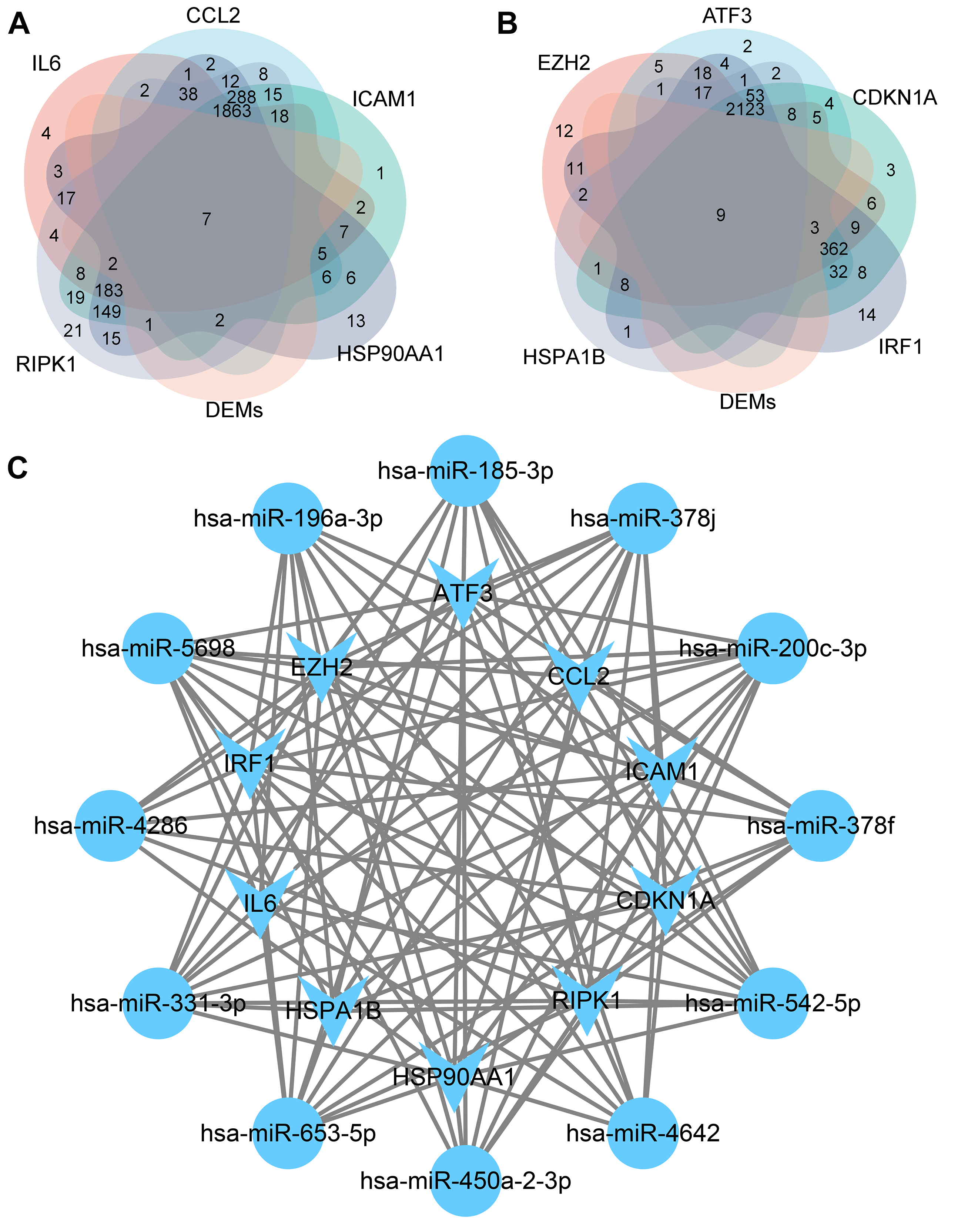


**Supplementary Figure S3. Construction of a miRNAs-mRNAs in PMD.** A: Venn analysis was conducted to predict the upstream PMD-related DEMs of hub genes IL6、CCL2、ICAM1、HSP90AA1、RIPK1. B: Venn analysis was conducted to predict the upstream PMD-related DEMs of ATF3、CDKN1A、IRF1、HSPA1B、EZH2. C: Cytoscape software was used to construct and visualize the miRNAs-mRNAs regulatory network.
